# Soil pH Filters the Association Patterns of Aluminum-Tolerant Microorganisms in Rice Paddies

**DOI:** 10.1128/msystems.01022-21

**Published:** 2022-02-15

**Authors:** Na Zhang, Zhiyuan Ma, Dong Li, Haowei Ni, Bo Sun, Yuting Liang

**Affiliations:** a State Key Laboratory of Soil and Sustainable Agriculture, Institute of Soil Science, Chinese Academy of Sciences, Nanjing, China; b University of Chinese Academy of Sciences, Beijing, China; Institute of Soil Science, Chinese Academy of Sciences

**Keywords:** aluminum toxicity, Al-tolerant bacteria, functional genes, directed network, niche breadth

## Abstract

Soil microbes are considered the second genome of plants. Understanding the distribution and network of aluminum (Al)-tolerant microorganisms is helpful to alleviate Al toxicity to plants in acidic soils. Here, we examined soluble Al^3+^ and bacterial communities carrying Al resistance genes in paddy soils with a soil pH range of 3.6 to 8.7. In the acidic soil with pH <5.1, the content of Al^3+^ increased significantly. There were abundant and diverse Al-tolerant microorganisms in acidic soils, including *Clostridium*, *Bacillus*, *Paenibacillus*, *Desulfitobacterium*, and *Desulfosporosinus*, etc. Moreover, compared with neutral and alkaline soils, the network structure of Al-tolerant microorganisms was more complex. The potential roles of major Al-tolerant microbial taxa on each other in the ecological network were identified by a directed network along 0.01 pH steps. The influential taxa in the network had a broader niche and contained more antioxidant functional genes to resist Al stress, indicating their survival advantage over the sensitive taxa. Our study is the first to explore the distribution of Al-tolerant microorganisms in continental paddies and reveal their potential associations mediated by pH, which provides a basis for further utilization of microbial resources in acidic agricultural soils.

**IMPORTANCE** Aluminum (Al) toxicity is the primary limiting factor of crop production in acidic soils with pH <5.0. Numerous studies have focused on the mechanism of Al toxicity and tolerance in plants; however, the effects of Al toxicity on soil microorganisms and their tolerance remain less studied. This study investigated the distribution and association patterns of Al-tolerant microorganisms across continental paddy fields with a soil pH range of 3.6 to 8.7. The results showed that soil pH filters exchangeable Al^3+^ content, diversity, and potential associations of Al-tolerant microbial community. The influential taxa in community network play an important role in Al tolerance and have potential applications in mitigating Al toxicity and promoting crop growth in acidic soils.

## INTRODUCTION

Acidic soils account for approximately 30% of global arable land, and their associated aluminum (Al) toxicity severely limits crop yields worldwide (1, 2). Al will be dissolved in the form of soluble Al^3+^ in the soil with pH <5.0, and a micromolar concentration of Al^3+^ in acidic soil will seriously interfere with the normal metabolic activities of soil biological communities and produce toxic effects on plants (3, 4). The continuous increase in soil acidity from anthropogenic activity and/or acid rain can further worsen the problem of Al toxicity and reduce agricultural productivity (5). The analysis of the existing literature shows that the current studies mainly focus on the mechanisms of Al tolerance in plants, including symplast tolerance and Al exclusion, while less attention has been paid to the Al tolerance of microorganisms (see Fig. S1 in the supplemental material).

10.1128/mSystems.01022-21.1 FIG S1 Researches on soil aluminum (Al), plants and microorganisms. We employ an advanced search term for topic OR title: (‘activated aluminum’ or ‘exchangeable aluminum’, or ‘Al^3+^’ and acidic soil); timespan: 1900 to 2020; databases: WOS, CSCD, CCC, DRCI, DIIDW, KJD, MEDLINE, RSCI, and SCIELO in Web of Science (articles: 2,030). Keywords related to (a) Al, plants, microorganisms and acidic soil, (b) Al, plants, and acidic soil, (c) Al, microorganisms, and acidic soil are selected to visualize the cooccurrence networks of researches focused on (a) soil Al with plants and microorganisms, (b) soil Al with plants, and (c) soil Al with microorganisms, respectively. The node and label sizes indicate the weight/frequency of the occurrence of text or terms. The higher the weight of an item, the larger the label and circle size of the item are. To avoid label overlap, the labels of certain items may not be displayed. The color of an item indicates the cluster containing the item, and the lines between items indicate links. Download FIG S1, TIF file, 2.9 MB. Copyright © 2022 Zhang et al. 2022 Zhang et al. https://creativecommons.org/licenses/by/4.0/ This content is distributed under the terms of the Creative Commons Attribution 4.0 International license.

Microorganisms, known as the second genome of plants, also have a variety of characteristics of Al toxicity resistance (6–8) that may play a vital role in alleviating plant growth stress. Microorganisms that can tolerant high concentrations of Al (for example, more than 100 μM or up to 200 mM) are termed Al-tolerant microorganisms. For example, *Rhodotorula* sp. strain RS1 can tolerate high levels of Al toxicity at 200 mM by thickening its cell wall (9). Some microorganisms secrete organic acid to chelate Al^3+^ outside their cells and thereby reduce Al toxicity (10). Cyanobacteria can induce antioxidant defense systems by increasing polyphenols, flavonoids, tocopherols, and glutathione levels as well as peroxidase, catalase, glutathione reductase, and glutathione peroxidase enzyme activities to resist Al-induced oxidative stress (11). It was also reported that Klebsiella species and *Serratia* species in rhizosphere soil can alleviate Al stress by forming complexes of Al^3+^ and siderophores (12). Most previous studies on Al-tolerant microorganisms and their mechanism of resistance to Al toxicity are focused on isolated superresistant microorganisms, especially fungi (such as Aspergillus flavus, *Penicillium* species, *Penicillium janthinellum*, and *Trichoderma asperellum*) and yeast strains (such as Cryptococcus *humicola*, *Rhodotorula glutinis*, and *Rhodotorula* species) (9, 13) as well as some bacterial strains (such as Pseudomonas fluorescens and *Burkholderia* species) (7, 14). Microorganisms harbor a diverse range of genes that encode Al resistance proteins. For example, *AANH_like*, *AAT_I*, and *Alr1p_like* are important genes encoding proteins and enzymes related to transport of Al^3+^ across membranes. *YnbB* encodes cystathionine beta-lyase family protein, which is involved in Al resistance by mediating inorganic ion transport and metabolism (Table S1) (15). To better explore Al-tolerant microbial resources and their potential ability to help crops reduce Al toxicity in acidic soils, it is necessary to study Al-tolerant microbes at the community level and clarify the factors affecting the community.

10.1128/mSystems.01022-21.6 TABLE S1 Al-resistant functional genes that encode Al resistance proteins detected by GeoChip 5.0 assay. Download Table S1, DOCX file, 0.02 MB. Copyright © 2022 Zhang et al. 2022 Zhang et al. https://creativecommons.org/licenses/by/4.0/ This content is distributed under the terms of the Creative Commons Attribution 4.0 International license.

Soil pH is one of the most important environmental factors influencing the diversity, interaction, activity, and function of the microbial community (16–18). The direct filtering effect of soil pH on microorganisms may be the consequence of their different optimal pH ranges for growth and activity (19, 20). Soil pH can affect soil characteristics, including Al^3+^ content and nutrient availability, which are important in shaping microbial community structure and associations (21). For example, the competition between bacteria and fungi changes along a continuous pH gradient (pH 4.0 to 8.3). This is due to the unfavorable bacterial physiology at low pH, which reduces bacterial fitness and, thus, enhances the growth of fungi (22). In turn, these changes may alter the functioning of the microbial community (23). The critical pH level at which reactive Al^3+^ increases notably and becomes toxic is 5.0 in most soils (3, 24). However, it is still unclear how soil pH alters the diversity and associations among Al-tolerant microorganisms. To develop Al-tolerant microbial resources suitable for growth and function under particular soil pH conditions, it is essential to investigate the diversity and association patterns of Al-tolerant microorganisms under a wide range of soil pHs.

Microbial network analysis has been widely used to explore microbial associations in complex environments (25–27). Although a cooccurrence network may not always reflect true ecological associations (28, 29), it can help to understand how the complexity of microbial community changes in response to environmental factors (30–32). Recently, a directed network has been used to infer the directionality of associations in ecology networks (33–35). In addition, the dependencies among the microbes in the community revealed by the directed network may have implications for their functions (34). Thus, it may provide an opportunity to understand the roles of different Al-tolerant microbial taxa in a community.

**FIG 1:**
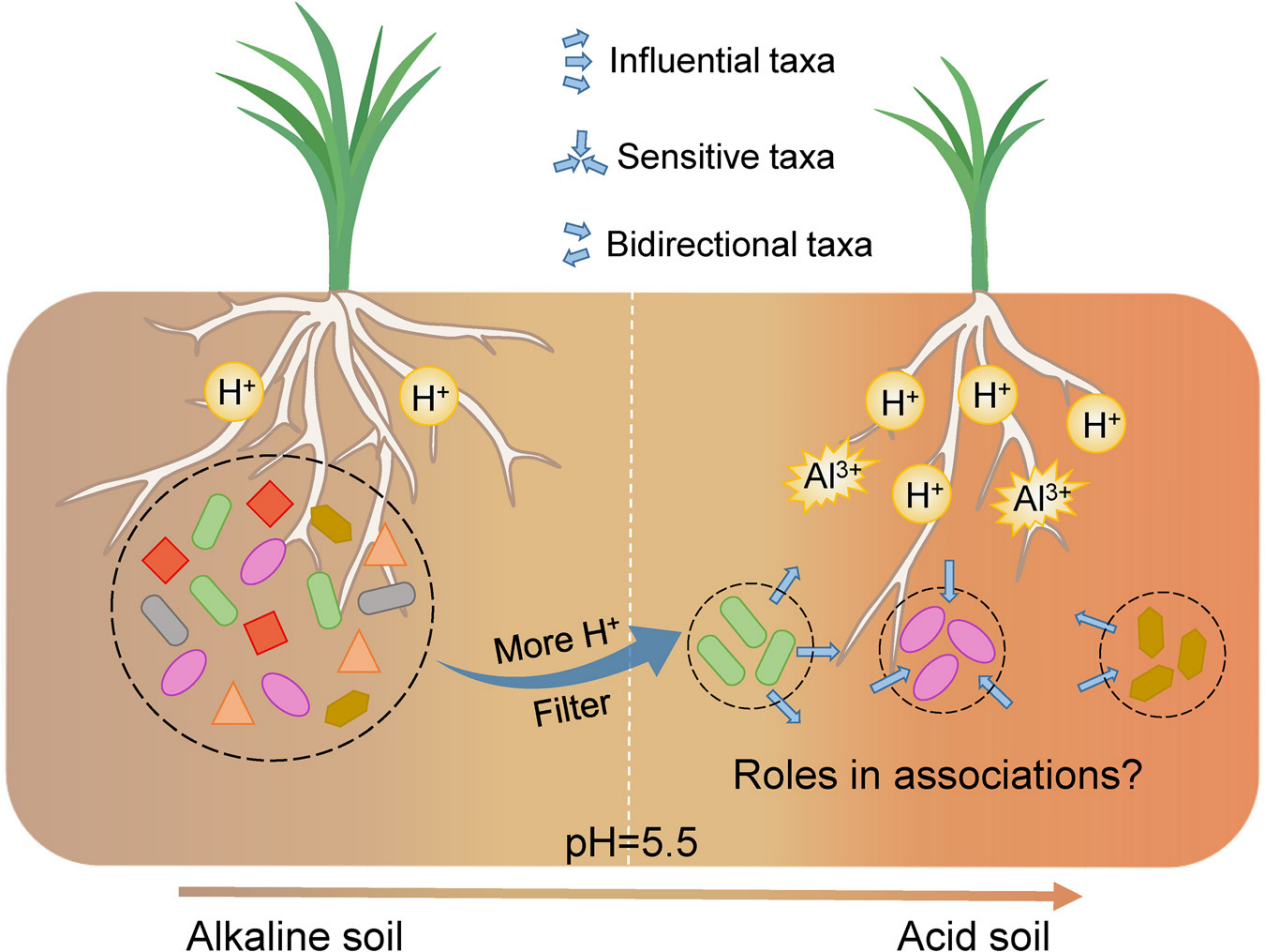
Hypothesis of this study. Reassembly of the soil microbial communities and prediction of the roles in microbial associations in acidic and highly exchangeable Al^3+^ soils.

In this study, we analyzed the distribution of soil Al^3+^ and Al-tolerant microbial communities across continental rice paddies with a wide soil pH range of 3.6 to 8.7. A directed network was constructed to explore the associations among Al-tolerant microorganisms on a continuous pH scale. We also determined the niche breadth and main influencing factors of different microbial roles in the associations. Here, we propose the following hypotheses (Fig. 1): (i) in rice paddy soils, pH filters the diversity and potential associations among Al-tolerant microorganisms and (ii) influential species in a community network play an important role in maintaining community-level Al tolerance in acidic rice paddy soils.

## RESULTS

### Distribution of Al-tolerant bacteria and functional genes in the rice fields.

**FIG 2:**
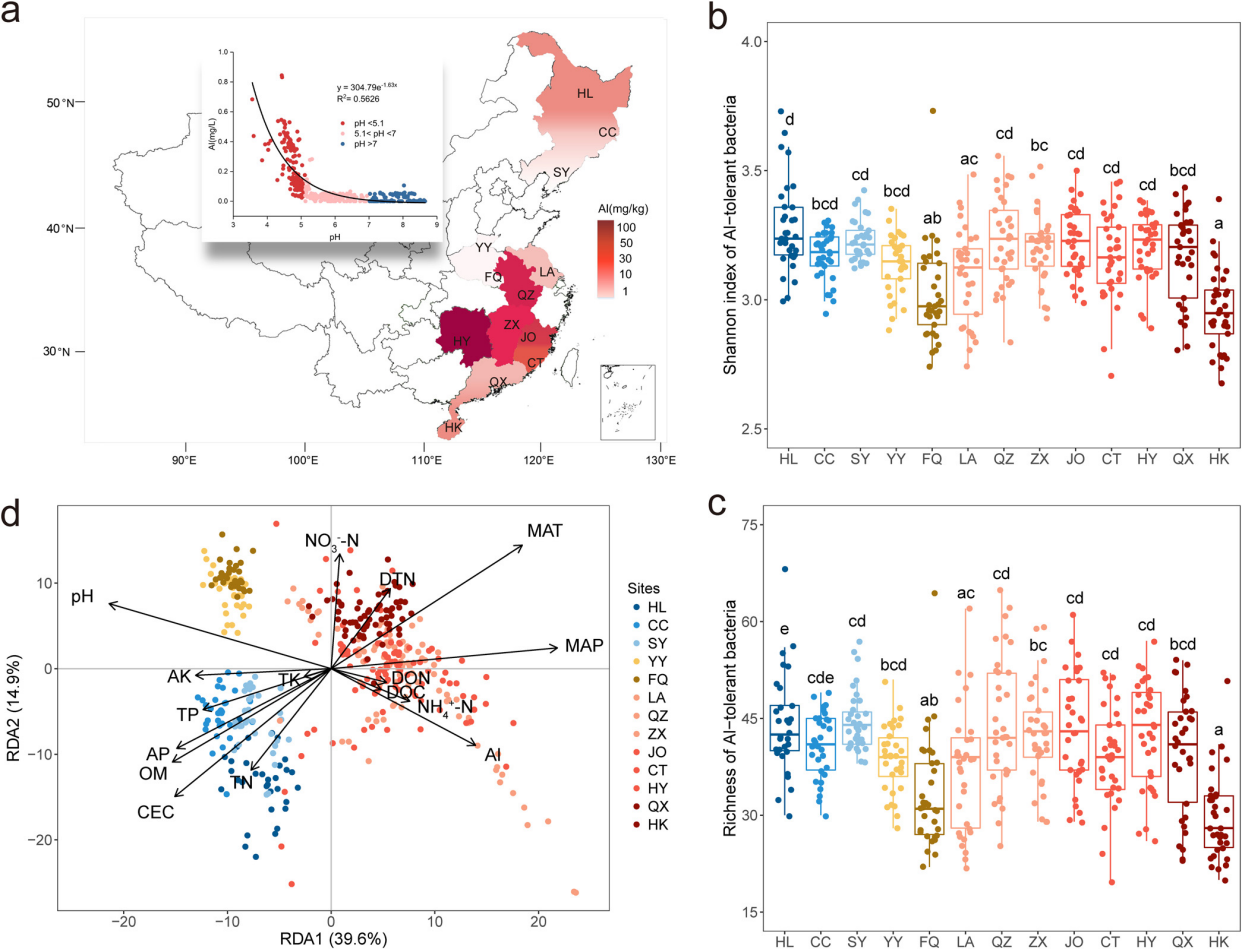
Distribution of Al-tolerant microorganisms in paddy soils. (a) Al content in 39 typical paddy fields from north to south China. (b and c) Shannon index (b) and richness (c) of Al-tolerant bacteria. The horizontal bars within the boxes indicate median values. The tops and bottoms of the boxes indicate the 75th and 25th percentiles, respectively. (d) Redundancy analysis of the Al-tolerant bacterial community structure. MAT, mean annual temperature; MAP, mean annual precipitation; DON, dissolved organic nitrogen; DTN, dissolved total nitrogen; TN, total nitrogen; OM, organic matter; DOC, dissolved organic carbon; TP, total phosphorus; AP, available phosphorus; TK, total potassium; AK, available potassium; CEC, cation exchange capacity.

Significant differences in exchangeable Al^3+^ content were observed in paddy soils from 13 regions in China from north to south (Fig. 2a). Al^3+^ content exponentially decreased with increasing soil pH (*R*^2^ = 0.56, *P < *0.01). The lower the soil pH value, the higher the content of Al^3+^ for pH ≤ 5.1. In addition, the content of exchangeable Al^3+^ was also related to soil properties such as soil organic matter, nitrogen, and phosphorus contents and climate conditions (see Fig. S2 in the supplemental material).

10.1128/mSystems.01022-21.2 FIG S2 Pearson correlations between Al^3+^ content and various environmental factors. MAT, mean annual temperature; MAP, mean annual precipitation; DON, dissolved organic nitrogen; DTN, dissolve total nitrogen; TN, total nitrogen; OM, organic matter; DOC, dissolved organic carbon; TP, total phosphorus; AP, available phosphorus; TK, total potassium; AK, available potassium; CEC, cation exchange capacity. Download FIG S2, TIF file, 1.9 MB. Copyright © 2022 Zhang et al. 2022 Zhang et al. https://creativecommons.org/licenses/by/4.0/ This content is distributed under the terms of the Creative Commons Attribution 4.0 International license.

**FIG 3:**
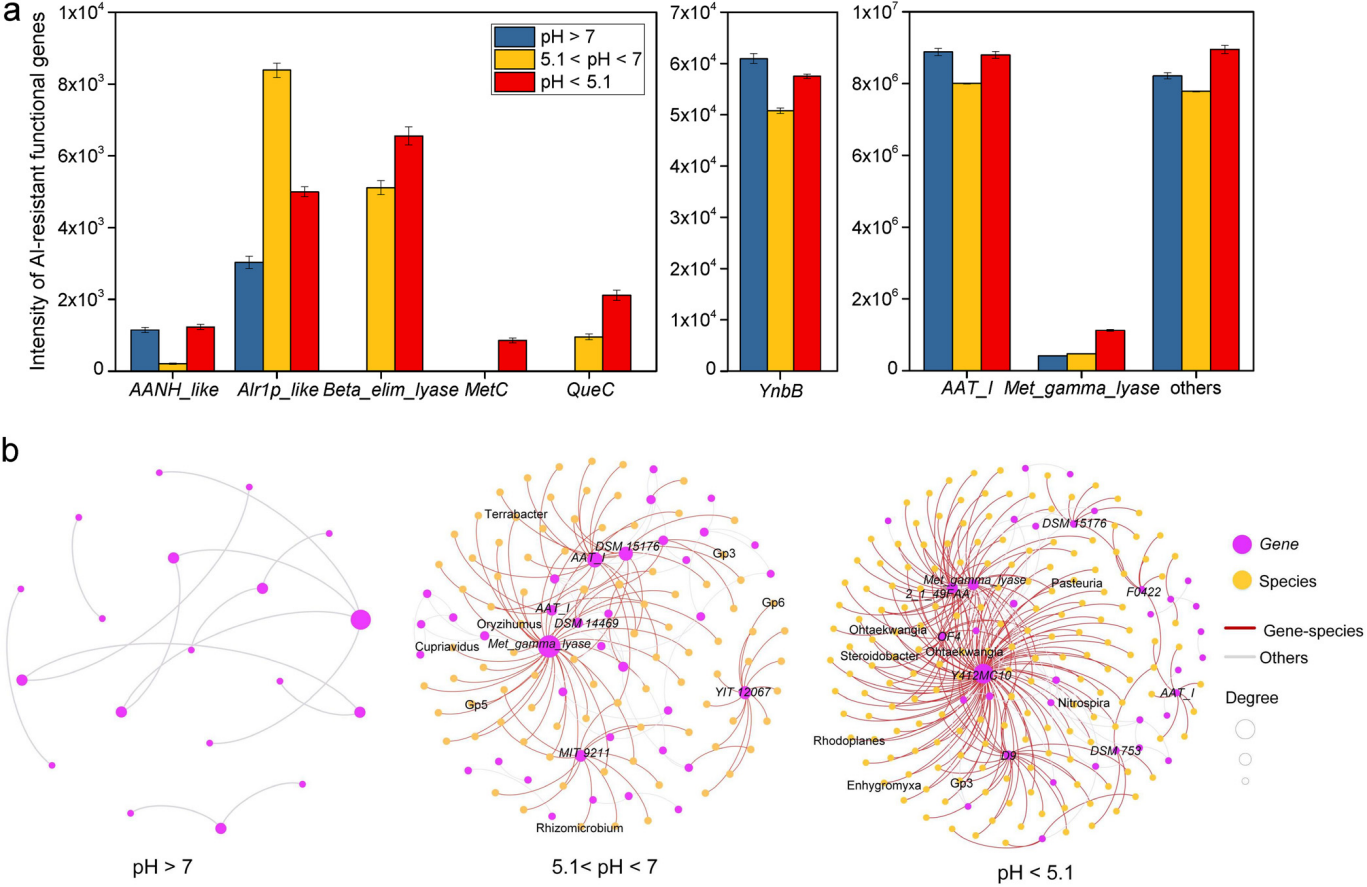
Associations between Al-tolerant microorganisms and Al-resistant genes in three pH ranges. (a) Intensity of Al-resistant functional genes at the different pH values. (b) Networks between Al-tolerant microorganisms and Al-resistant functional genes at different pH values. A connection indicates a strong (Spearman’s *r* > 0.6) and significant (false discovery rate-corrected *P < *0.01) correlation. The size of each node is proportional to the degree; the thickness of a connection between two nodes (i.e., an edge) is proportional to the value of Spearman’s correlation coefficient. Yellow indicates the bacterial OTUs, and purple indicates the Al-resistant functional genes. Some Al-resistant functional genes without specific region names were labeled with their potential source strains, which belong to the other Al-resistant functional genes. The red lines indicate the connections between genes and species, and the gray lines indicate the connections between genes and genes and/or species and species.

Al-tolerant bacterial communities were identified according to the microorganisms carrying Al-resistant functional genes detected by GeoChip 5.0 (36). In total, 233 unique Al-resistant functional genes were detected by GeoChip 5.0 (Table S1), and 233 source organisms were considered potential Al-tolerant microorganisms, belonging to 92 genera (Table S2). Finally, 461 operational taxonomic units (OTUs) of 55,519 OTUs (0.83%) detected by 16S were found to belong to these 92 genera. These OTUs were inferred to be Al-tolerant microorganisms for subsequent analysis. The Shannon index and richness of Al-tolerant bacteria varied across the sampling area (Fig. 2b and c). Unexpectedly, the Shannon index and richness of Al-tolerant bacteria were not significantly higher at sites of higher Al content. A possible explanation is that Al content may not be the most important driving factor of Al-tolerant bacterial communities. Redundancy analysis indicated a clear biogeographic distribution pattern of Al-tolerant bacteria (Fig. 2d). The community structure of Al-tolerant bacteria was influenced by mean annual temperature (MAT), mean annual precipitation (MAP), soil pH, cation exchange capacity (CEC), organic matter (OM), available phosphorus (AP), and exchangeable Al^3+^ (*P = *0.001). Partial Mantel analysis also suggested that soil pH was the most important soil factor affecting the Al-tolerant bacterial community (Table S3). Similarly, the abundance of microbial Al-resistant genes varied with soil pH, with more functional genes that encode Al resistance protein, such as *AANH_like*, *Beta_elim_lyase*, *MetC*, *QueC*, *Met_gamma_lyase*, and others, showing higher abundance at pH <5.1 than those at pH >7 and 5.1 < pH < 7 (Fig. 3a, Table S1). Since resistance to metal oxidative stress is an important mechanism for microbial tolerance to Al toxicity in acidic soils, we also analyzed the diversity of microbial antioxidant functional genes, including *cat_arc*, *cat_bac*, *cat_fun*, *per_arc*, *per_bac*, *per_fun*, *sod_CuZn*, *sod_FeMn*, and *sod_nickel* (Table S4). The highest levels of antioxidant gene richness and abundance were found in soils with pH <5.1, followed by soils with 5.1< pH < 7, while soils with pH >7 showed the lowest levels of richness and abundance (Fig. S3a). The abundances of *AAT_I*, *Met_gamma_lyase*, and other Al-resistant genes were significantly correlated with the abundances of these antioxidant genes (*r* = 0.30 to 0.81, *P < *0.001), while the abundance of *Alr1p_like* was positively correlated with that of *cat_arc*, *cat_bac*, *cat_fun*, *per_arc*, *per_bac*, and *per_fun* (*P < *0.05) and the abundance of *YnbB* was related to that of *cat_bac*, *per_bac*, *per_fun*, *sod_CuZn*, and *sod_FeMn* (*P < *0.05) (Fig. S3b).

10.1128/mSystems.01022-21.3 FIG S3 (a) Alpha diversity of the antioxidant functional genes. (b) Spearman correlation between the abundance of Al-resistant genes and antioxidant genes. The color gradient denoting Spearman’s correlation coefficient. *, **, and *** indicate *P < *0.05, *P < *0.01, and *P < *0.001, respectively. Download FIG S3, TIF file, 0.4 MB. Copyright © 2022 Zhang et al. 2022 Zhang et al. https://creativecommons.org/licenses/by/4.0/ This content is distributed under the terms of the Creative Commons Attribution 4.0 International license.

10.1128/mSystems.01022-21.7 TABLE S2 Taxonomic information of source organisms of Al-resistant functional genes detected by GeoChip 5.0. Download Table S2, DOCX file, 0.01 MB. Copyright © 2022 Zhang et al. 2022 Zhang et al. https://creativecommons.org/licenses/by/4.0/ This content is distributed under the terms of the Creative Commons Attribution 4.0 International license.

10.1128/mSystems.01022-21.8 TABLE S3 Partial Mantel analysis of the relationship between the environmental variables and Al-tolerant bacterial community. Download Table S3, DOCX file, 0.01 MB. Copyright © 2022 Zhang et al. 2022 Zhang et al. https://creativecommons.org/licenses/by/4.0/ This content is distributed under the terms of the Creative Commons Attribution 4.0 International license.

10.1128/mSystems.01022-21.9 TABLE S4 Antioxidant functional genes on GeoChip 5.0 array. Download Table S4, DOCX file, 0.01 MB. Copyright © 2022 Zhang et al. 2022 Zhang et al. https://creativecommons.org/licenses/by/4.0/ This content is distributed under the terms of the Creative Commons Attribution 4.0 International license.

The network between Al-tolerant bacteria and Al-resistant genes showed different association patterns across the three pH ranges (Fig. 3b). The network was most complex at pH <5.1 with strong connections between bacterial taxa and Al-resistant genes. In contrast, at pH >7, Al-tolerant bacteria and Al-resistant genes were rarely linked. These results suggest that soil pH is a key factor in determining the diversity of Al-tolerant bacteria and Al-resistant genes and their association patterns, and that the diversity of Al-tolerant bacteria and Al-resistant genes is the highest and the association is the strongest at pH <5.1.

### Directed network to distinguish the different roles of microorganisms.

**FIG 4:**
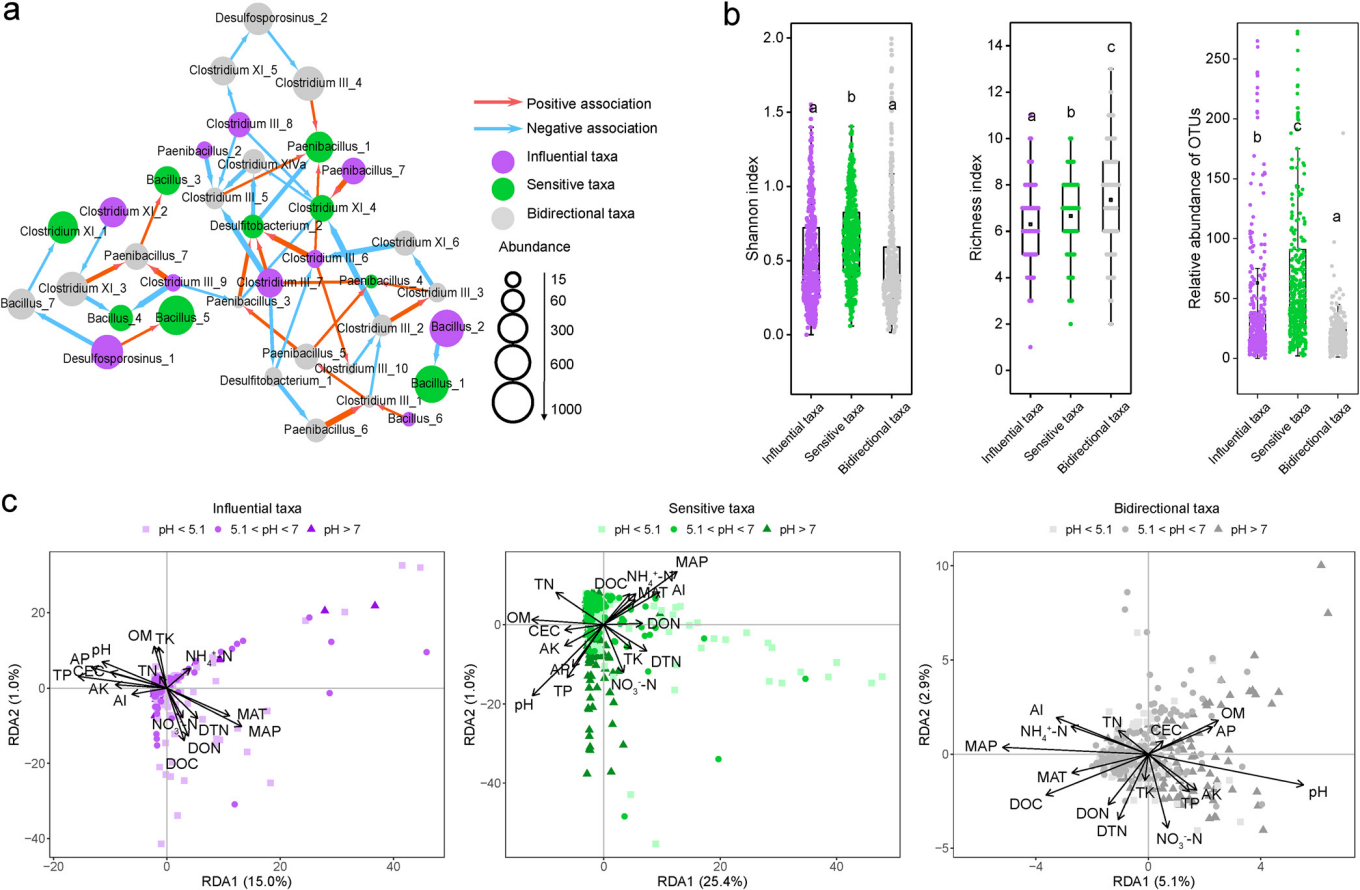
Roles and diversity of the three microbial taxa in the directed network. (a) Inferred potential microbial interdependent associations in acidic soils. The nodes are the OTUs labeled with their genus-level taxon name, and the directed edges are the nonzero entries in the inferred interaction matrix. The directed positive and negative edges are colored in red and blue, respectively. Orphan nodes are not shown. (b) Alpha diversity (Shannon index, richness, and relative abundance) of three functional taxa. The horizontal bars within the boxes indicate median values. The tops and bottoms of the boxes indicate the 75th and 25th percentiles, respectively. (c) Redundancy analysis of community compositions of three functional taxa. MAT, mean annual temperature; MAP, mean annual precipitation; DON, dissolved organic nitrogen; DTN, dissolved total nitrogen; TN, total nitrogen; OM, organic matter; DOC, dissolved organic carbon; TP, total phosphorus; AP, available phosphorus; TK, total potassium; AK, available potassium; CEC, cation exchange capacity.

To further explore the potential roles of Al-tolerant microorganisms under stress from low pH, we constructed a directed network that could infer their potential interactive relationships. First, we used four types of noise (black, brown, pink, and white noise) to evaluate the dependence of microbial communities within each pH range, which ranges from black in case of the strongest dependency to white in the absence of any dependency (Fig. S4). The higher percentage (50%) of non-white noise types indicated a stronger dependence between bacteria in the continuous series of pH <5.1. According to the in-degree (the number of associations pointing to the node, indicating the degree of the species affected by others) and out-degree (the number of associations pointing out of the node, indicating the degree of the species affecting others) of the directed network in the acidic soil (pH < 5.1), the Al-tolerant microorganisms were inferred to be divided into three functional taxa: the influential taxa (out-degree only), the sensitive taxa (in-degree only), and the bidirectional taxa (both out-degree and in-degree) (Fig. 4a, Table S5). These microorganisms mainly belong to the genera *Clostridium*, *Bacillus*, *Paenibacillus*, *Desulfitobacterium*, and *Desulfosporosinus* (Table S5). The Shannon diversity of the sensitive taxa and relative abundance of the influential and sensitive taxa were higher than those of the bidirectional taxa, while the bidirectional taxa showed higher richness (*P < *0.05) (Fig. 4b).

10.1128/mSystems.01022-21.4 FIG S4 Noise-type profile distinguishes dependence between successive pH steps occurred in the pH series. A shift in the noise color from black to pink indicates a reduced dependency on community within the pH series, while white noise indicates the absence of any dependency. In brief, the dependency on community between pH steps is strongest for black noise and weakest for pink noise, while it is absent for white noise. Download FIG S4, TIF file, 0.2 MB. Copyright © 2022 Zhang et al. 2022 Zhang et al. https://creativecommons.org/licenses/by/4.0/ This content is distributed under the terms of the Creative Commons Attribution 4.0 International license.

10.1128/mSystems.01022-21.10 TABLE S5 Classification of three taxa based on the out- and in-degrees of the directed network in acidic soils (pH < 5.1). Download Table S5, DOCX file, 0.02 MB. Copyright © 2022 Zhang et al. 2022 Zhang et al. https://creativecommons.org/licenses/by/4.0/ This content is distributed under the terms of the Creative Commons Attribution 4.0 International license.

As shown by the nonmetric multidimensional scaling (NMDS) ordination of the communities (Table 1, Fig. S5), MAP, and pH explained more variations in influential community structure (12.90% by MAP and 11.90% by pH). dissolved organic carbon (DOC) and Al^3+^ contributed more to the sensitive community structure (12.30% by DOC and 8.33% by Al^3+^). The community structure of bidirectional taxa was explained most by MAP (18.30%), exchangeable Al^3+^ (15.20%), and pH (12.50%).

**TABLE 1:** Role of environmental variables in determining the community composition of three functional taxa as measured with GAMs^*a*^

Variable	Influential taxa		Sensitive taxa		Bidirectional taxa	
	Deviance explained (%)	Significance	Deviance explained (%)	Significance	Deviance explained (%)	Significance
MAT	5.03	0.003	2.73	>0.05	10.20	<0.001
MAP	12.90	<0.001	6.84	0.001	18.30	<0.001
Exchangeable Al^3+^	2.69	0.003	8.33	<0.001	15.20	<0.001
pH	11.90	<0.001	6.44	0.005	12.50	<0.001
NH_4_^+^-N	4.35	>0.05	2.53	>0.05	3.00	>0.05
NO_3_-N	3.76	>0.05	1.88	>0.05	7.43	0.004
DON	4.94	>0.05	2.42	>0.05	1.83	0.019
DTN	4.08	>0.05	1.32	>0.05	5.58	0.030
TN	6.48	<0.001	5.13	>0.05	5.89	0.016
OM	3.64	<0.001	4.46	0.024	7.58	<0.001
DOC	4.47	>0.05	12.30	<0.001	1.85	0.019
TP	2.60	0.004	1.60	>0.05	3.75	0.044
AP	6.50	0.007	4.45	>0.05	9.14	<0.001
TK	2.76	>0.05	2.13	>0.05	0.63	>0.05
AK	6.87	<0.001	1.07	>0.05	5.64	<0.001
CEC	7.45	<0.001	1.37	>0.05	6.96	0.004

a MAT, mean annual temperature; MAP, mean annual precipitation; DON, dissolved organic nitrogen; DTN, dissolve total nitrogen; TN, total nitrogen; OM, organic matter; DOC, dissolved organic carbon; TP, total phosphorus; AP, available phosphorus; TK, total potassium; AK, available potassium; CEC, cation exchange capacity.

10.1128/mSystems.01022-21.5 FIG S5 Results of the generalized additive models fitting measured environmental data across the nonmetric multidimensional scaling (NMDS) ordination of the measured (a) influential taxa, (b) sensitive taxa, and (c) bidirectional taxa. The blue splines indicate the fit of the environmental data from high values (dark blue) to low values (light blue) over the ordination. The environmental overlay indicates that communities, as represented by the points on the NMDS, are associated with higher or lower environmental values, in line with the colored environmental gradient. Note that the gradient splines are parallel if the relationship between the environment and community is linear. The nonlinear relationships between the environment and community composition are indicated as curved splines. de shows the deviance explained by the respective model. MAT, mean annual temperature; MAP, mean annual precipitation; DON, dissolved organic nitrogen; DTN, dissolve total nitrogen; TN, total nitrogen; OM, organic matter; DOC, dissolved organic carbon; TP, total phosphorus; AP, available phosphorus; TK, total potassium; AK, available potassium; CEC, cation exchange capacity. Download FIG S5, TIF file, 2.8 MB. Copyright © 2022 Zhang et al. 2022 Zhang et al. https://creativecommons.org/licenses/by/4.0/ This content is distributed under the terms of the Creative Commons Attribution 4.0 International license.

### Niche breadth and potential resistance of three taxa of Al-tolerant microorganisms.

**FIG 5:**
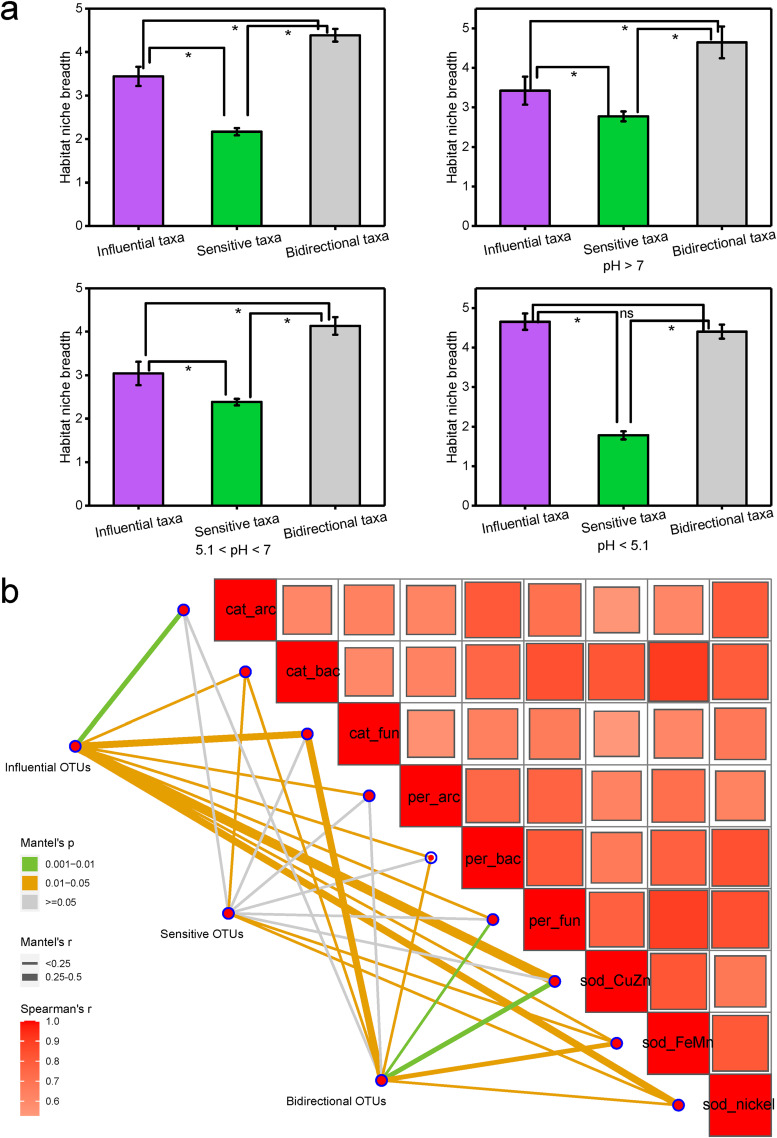
Niche breadth of three functional taxa and their relationship with antioxidant functional genes. (a) Comparison of mean habitat niche breadths (Bcom) of different functional taxa in bacterial community in all samples and in samples with different pH conditions (nonsignificant [n.s.], *P > *0.05; *, *P < *0.05; Duncan’s test). (b) Pairwise comparisons of the antioxidant function genes are shown, with a color gradient denoting the Spearman correlation coefficient. The taxonomic community composition is related to each antioxidant function gene via partial Mantel tests. The edge width corresponds to Mantel’s *r* statistic for the corresponding distance correlation, and the edge color denotes the statistical significance based on 9,999 permutations.

Niche analysis explained the different responses of the three taxa in the Al-tolerant bacterial community. The mean community-level *B* value (Bcom) values for sensitive taxa were significantly lower under all pH conditions compared to the other two taxa, suggesting that sensitive taxa typically occupy the smallest niche breadths in the environment (Fig. 5a). Notably, the niche breadths of the influential and bidirectional taxa were 23.4% and 67.7% larger than that of the sensitive taxa at pH >7, 27.7% and 73.6% larger at 5.1 < pH < 7, and 161.7% and 147.7% larger at pH <5.1. The difference between the niche breadths of the sensitive taxa and the influential and bidirectional taxa was greater at pH <5.1 than at pH >5.1. The niche breadth of sensitive taxa at pH <5.1 was significantly smaller than those at 5.1 < pH < 7 and at pH >7 (*P < *0.05) (Table 2), suggesting that stress from low pH further narrows the niche width of sensitive taxa. In contrast, influential and bidirectional taxa may be less affected or even promoted because of their greater niche breadth. Major climatic and edaphic variables explained more variation of the sensitive taxa community composition (26.4% with the first two axes) than that of the influential taxa (16.0% with the first two axes) and the bidirectional taxa (8.0% with the first two axes) (Fig. 4c). The community composition of the sensitive taxa was more affected by stress from low pH (*r*^2^ = 20.3%, *P < *0.001) than the influential taxa (*r*^2^ = 6.6%, *P < *0.001) and the bidirectional taxa (*r*^2^ = 18.9%, *P < *0.001).

**TABLE 2:** Bcom of different functional taxa in samples under different pH conditions^*a*^

Habitat niche breadth	Influential taxa	Sensitive taxa	Bidirectional taxa
pH >7	3.42 ± 0.36 b	2.77 ± 0.13 c	4.64 ± 0.40 b
5.1 < pH < 7	3.04 ± 0.27 a	2.38 ± 0.08 b	4.13 ± 0.20 a
pH <5.1	4.65 ± 0.21 c	1.78 ± 0.10 a	4.40 ± 0.18 b

a The different letters indicate that niche breadths of the influential taxa, the sensitive taxa, or the bidirectional taxa are significantly different (Duncan’s test at *P *< 0.05) under different pH conditions.

To further understand the mechanism of Al tolerance, we linked distance-corrected differences in community composition of the three taxa to differences in microbial antioxidant genes (Fig. 5b). Overall, influential taxa showed the strongest linkages with antioxidant genes, including *cat_arc*, *cat_bac*, *cat_fun*, *per_arc*, *per-bac*, *per_fun*, *sod_CuZn*, *sod_FeMn*, and *sod_nickel*. Sensitive taxa only correlated with *cat_bac*, *sod_FeMn*, and *sod_nickel*. These results suggest that antioxidant gene composition is more relevant to the community structure of influential taxa and less related to sensitive taxa.

## DISCUSSION

Our study of Al-tolerant microbes in the continental-scale paddy soils showed that soil pH was the most important factor driving the diversity and association patterns of Al-tolerant bacterial communities. As expected, Al-tolerant microorganisms in acidic soils with high Al content exhibited a more complex network. Microorganisms with similar habitat and environmental preferences (e.g., low pH or high pH) tend to coexist in ecosystems (37, 38). Our results suggested distinct association patterns of Al-tolerant bacteria and functional genes in three different pH ranges, implying the contrasting habitat preferences of bacteria in paddy fields across continental scales. The associations among Al-tolerant bacteria were the most complex in soil with pH <5.1, which are mainly distributed in southern China. These may result from more Al-tolerant microbial populations induced in acidic soils through dispersal or evolutionary processes. It was reported that some microorganisms may adapt to soil acidification through evolution, and endemic species already adapted to the acidic pressure can colonize the acidic soils through dispersal (39). Similarly, long-term high exchangeable Al^3+^ concentration in acidic soils may enhance bacterial tolerance to Al toxicity, whereas non-Al-tolerant bacteria dominated under neutral or weakly alkaline conditions. Another possible explanation is that these Al-tolerant microbial populations exhibit more functional interdependence in acidic environments. To survive under the poor soil condition with low pH, nutrient deficiencies, and high Al toxicity, more associations among species may help them to perform functions better, such as participating in nutrient cycling and mitigating toxicity (40).

Different functional taxa of soil microorganisms may show different interdependencies and establish complex networks of interactions due to their specific functional traits (41). Environmental stresses can collectively affect various microbial functions to resist selective stresses (42). In turn, the function of microbial communities affects their environment, leading to further changes in associations with its members (43). For example, resistant microorganisms may coexist to relieve environmental stress and provide favorable conditions for the survival of sensitive microorganisms (44). As we inferred from the directed network, the influential and bidirectional taxa may have effects on sensitive taxa and benefit them under acidic stress. In microbial community dynamics, species always evolve with other species and adapt not only to the environment itself but also to the biological environment formed by other species (43). Here, different microbial functional roles may be determined in part by their different ecological niches. The influential and bidirectional taxa occupy multiple niches and can use more resources to promote their growth under acidic stress. In contrast, the sensitive taxa with narrow niche breadth may be more affected by low pH. This finding is consistent with previous observations that habitat generalists with wider niche breadth were generally less affected by environmental factors (45). The distributions of influential and bidirectional taxa may remain more stable than those of sensitive taxa when microbial communities are disturbed by environmental stress, resulting in advantages in complementary resource acquisition and stress tolerance (46, 47).

Environmental filtration regulates the connections between microbial communities, thereby affecting their roles and ecological functions (38, 48). In life history strategies, certain traits tend to be interrelated due to physiological or evolutionary tradeoffs, and different strategies are adopted under different environmental conditions (47). In this study, most of the influential species in acidic soils belonged to genus *Clostridium*, while most of the sensitive species belonged to genera *Bacillus* and *Clostridium*. Aerobic *Bacillus* and anaerobic *Clostridium* are two genera of Gram-positive bacteria belonging to the phylum *Firmicutes* (49, 50). Sporulation is an important strategy for *Clostridium* and *Bacillus* species to survive longer in many environmental niches, especially under stressed conditions (51). *Bacillus* and *Clostridium* have been shown to be closely related in their sporulation mechanism, i.e., the regulatory protein Spo0A and its downstream pathways (52). Although the activation of the Spo0A pathway is different between the two taxa (53, 54), *Clostridium* species may complement the Al tolerance of *Bacillus* species and help them survive in acidic paddy fields.

Another possible strategy to improve acid and Al tolerance of organisms is to manipulate antioxidant defense system (55, 56). Al-induced accumulation of reactive oxygen species and changes in cell wall properties are considered two intrinsic factors in Al toxicity (57, 58). We found that the influential taxa were strongly correlated with multiple antioxidant functional genes, while the sensitive taxa were weakly correlated with these genes. It implies that antioxidant genes tend to be possessed more by influential taxa than sensitive taxa. For example, superoxide dismutase (SOD) encoded by *sod_CuZn*, *sod_FeMn*, and *sod_nickel* genes is a universal enzyme involved in regulating intracellular reactive oxygen species and protecting cells from the exogenous toxicity of oxidants (59).

As the second genome of plants, Al-tolerant microorganisms can alleviate Al toxicity in acidic soils and improve the tolerance of plants to Al stress. Al-tolerant microorganisms may reduce soil Al toxicity by absorbing, adsorbing, and secreting organic acids to chelate Al^3+^ (10). Some Al-tolerant microorganisms, such as Pseudomonas simiae N3, Chryseobacterium polytrichastri N10, and Burkholderia ginsengiterrae N11-2, can increase the plant tolerance against Al stress by inducing expression of Al stress-related genes (*AtAIP*, *AtALS3*, and *AtALMT1*) (60). In addition to tolerance to high Al concentration, Klebsiella sp. strain RC3, *Stenotrophomonas* sp. strain RC5, Klebsiella sp. strain RCJ4, *Serratia* sp. strain RCJ6, and Enterobacter sp. strain RJAL6 exhibit multiple plant growth-promoting traits (P solubilization, indole acetic acid production, 1-aminocyclopropane-1-carboxylate deaminase activity, and exudation of organic acid anions and siderophores) (12, 61). Moreover, some Al-tolerant microorganisms may support the growth of some sensitive microorganisms that carry out functions essential to plant growth in acidic soil. As microbial associations revealed by the directed network, the influential and bidirectional taxa may provide benefits to the sensitive taxa. Thus, diverse Al-tolerant microorganisms and their potential associations can be used in the future for the new approach to promote plant resistance and growth in acidic soils.

In conclusion, our study of Al-tolerant bacterial communities across continental paddy soils revealed that soil pH is a major determinant of exchangeable Al^3+^ content as well as diversity and association patterns of Al-tolerant microbial communities. Al-tolerant microorganisms exhibited the most complex network structure and potential functional interdependence in acidic soil of pH <5.1. For the first time, we provide a continental distribution of Al-tolerant microorganisms at the community level. By constructing a directed network, potential interdependence of microorganisms in stress tolerance were inferred from the functional relationships among influential, sensitive, and bidirectional taxa. Further analysis suggests that different microbial functional roles in the associations are driven in part by niche differences among microbes. The functional taxa identified from the microbial communities have potential applications in the development of microbial resources to mitigate Al toxicity and promote crop growth. This study provides a basis for further utilization of microbial resources in acidic agricultural soils. If the plant growth-promoting bacteria are sensitive to Al, they may be protected in the community by other highly resistant species. The tolerance of rhizosphere and plant microbiota to Al stress may be improved by adjusting the proportion of influencing taxa.

## MATERIALS AND METHODS

### Site description and sampling.

A total of 429 soil samples were obtained in 2013 from 39 paddy fields in 13 regions along a north-south transect across eastern China (110°41'E to 126°92'E, 19°76'N to 47°58'N): Hailun (HL), Changchun (CC), Shenyang (SY), Yuanyang (YY), Fengqiu (FQ), Linan (LA), Quzhou (QZ), Zixi (ZX), Jianou (JO), Changting (CT), Henyang (HY), Qingxin (QX), and Haikou (HK) (Fig. 2a). The sampled paddy fields represented four types of cropping systems (single cropping of rice, rice-wheat rotation, double cropping of rice, and triple cropping of rice) and five soil types (neutral black soil derived from loamy loess, alkaline fluvo-aquic soil derived from Yellow River alluvial sediments, hydromorphic paddy soil derived from lake sediments, acidic red soil derived from Quaternary red clay, and submergenic paddy soil derived from neritic sediments). Three paddy fields with similar inorganic fertilizations and irrigation practices were selected in each region. Surface (0 to 20 cm) soil samples were collected from paddy fields after the rice harvest for single-season rice and rice-wheat rotations and after the late-season rice harvest for two-season rice and three-season rice crops. At each sampling field, 11 soil samples were collected within 100- by 100-m plots based on a spatially explicit L-shaped sampling design, with distances between the adjacent subplots of 0, 1, 5, 10, 20, and 40 m, respectively. Five cores with a diameter of 5 cm were randomly selected from the topsoil layer (0 to 20 cm), and each subsample point was 0.5 m in diameter and mixed together (500 g in total). The soil samples were collected and sealed in sterile sampling bags and transported to the laboratory on ice. In the laboratory, soil samples were sieved to 2 mm to remove visible roots and residues, homogenized, and subdivided into two subsamples. One subsample was stored at 4°C to measure geochemical properties, and the other was stored at −80°C for molecular analyses. The experiments for measuring soil geochemical properties and microbial molecular analyses, including soil microbial DNA extraction, 16S rRNA gene amplicon sequencing, and GeoChip 5.0 hybridization, were carried out within half a year after soil sampling.

Soil geochemical properties were measured according to the recommended testing procedures (62). Soil exchangeable Al^3+^ was measured by extraction with 1 mol/liter potassium chloride (KCl) solution (1:50, wt/vol). Soil suspensions were shaken at 180 rpm for 30 min, centrifuged at 3,000 rpm for 10 min, and then filtered. Al concentration in soil solution was determined by inductively coupled plasma atomic emission spectrophotometry (ICP-OES; Optima 8000; PerkinElmer, USA) after appropriated dilution. Soil pH was determined with a glass electrode at a water-to-soil ratio of 2.5:1. Soil OM and DOC contents were measured using the Walkley-Black wet oxidation method (63). Soil CEC was measured based on the ammonium acetate saturation (AMAS) method (64). TN, NO_3_-N, and NH_4_^+^-N contents were determined by the Kjeldahl method (65). Dissolved total nitrogen (DTN) was determined by alkaline persulfate oxidation, and dissolved organic nitrogen (DON) was calculated as DON = DTN – (NH_4_^+^-N) – (NO_3_-N). TP and AP contents were digested with sulfuric acid and perchloric acid (H_2_SO_4_-HClO_4_), extracted by sodium carbonate and sodium bicarbonate, respectively, and then measured using the molybdenum-blue method (66). Total potassium (TK) and AK contents were measured by flame photometry (FP6400A; CANY Precision Instrument Co., Ltd., Shanghai, China) after extraction with sodium hydroxide and ammonium acetate, respectively (67). Climatic data, including MAT and MAP, were obtained from the WorldClim database (www.worldclim.org). All soil geochemical data are available in the figshare repository (https://doi.org/10.6084/m9.figshare.11493081.v2).

### Soil microbial DNA extraction and 16S rRNA gene amplicons sequencing.

For each sample, microbial genomic DNA was extracted from 2 g of well-mixed soil by combining the cryogenic grinding method and SDS for cell lysis, as described previously (68). Crude DNA was further purified by agarose gel electrophoresis, followed by successive extractions with phenol, chloroform, and butanol (68, 69). The concentration and quality of the purified DNA was determined by an ND-1000 spectrophotometer (NanoDrop, Inc., Wilmington, DE, USA) and a Quant-It PicoGreen kit (Invitrogen, Carlsbad, CA, USA). The bacterial V4 region of the 16S rRNA genes was amplified with the common primer set 515F (5′-GTGCCAGCMGCCGCGGTAA-3′) and 806R (5′-GGACTACHVGGGTWTCTAAT-3′) (70). The forward and reverse primers were tagged with adapters, pads, and linker sequences. Each barcode sequence (12 mer) was added to the reverse primer for pooling of multiple samples in one run of MiSeq sequencing. PCR amplification was performed in triplicate with a GeneAmp PCR system 9700 (Applied Biosystems, Foster City, CA, USA) in a 25-μl reaction system that consisted of 2.5 μl of 10× PCR buffer II and 0.5 U of Herculase II DNA polymerase high fidelity (Agilent, USA), 1 μl (10 μM) of each primer, and 15 μl of template DNA. PCR was performed to target the 16S rRNA gene through cycling conditions of initial denaturation at 94°C for 1 min, followed by 30 cycles at 94°C for 20 s, 53°C for 25 s, and 68°C for 45 s, with a final extension at 68°C for 10 min. The PCR products from the three replicates were combined and purified with an Agencourt AMPure XP kit (Beckman Coulter, Brea, CA, USA). The PCR products were examined by electrophoresis with 1% agarose gel and quantified by PicoGreen with FLUOstar Optima (BMG Labtech, Jena, Germany). The PCR products were pooled from different samples in equal amounts and then purified using the Qiagen gel extraction kit (Qiagen Sciences, Germantown, MD, USA) in accordance with the manufacturer’s instructions and requantified by PicoGreen.

The sequencing samples were prepared using a TruSeq DNA kit in accordance with the manufacturer’s instructions. The purified library was diluted, denatured, rediluted, and mixed with PhiX (equal to 30% of the final DNA amount) as described in the Illumina library preparation protocols. Afterward, the purified library was applied to an Illumina MiSeq system at the Institute for Environmental Genomics at the University of Oklahoma for sequencing with the reagent kit v2, 2 × 250 bp, as described in the manufacturer’s manual (Illumina, San Diego, CA, USA).

After assigning each sequence to its sample according to its barcode, 6,139,308 reads for bacteria were obtained for all 429 samples. The sequence data were processed using the QIIME Pipeline version 2 (http://qiime.org/). All sequence reads were trimmed and assigned to each sample on the basis of their barcodes. The sequences with high quality (length of >200 bp, without ambiguous base N, and average base quality score of >30) were used for downstream analysis. Operational taxonomic units (OTUs) were clustered using the recently introduced program UPARSE at 97% similarity level (71). Final OTUs were generated using the clustering results, and taxonomic annotations were assigned to each OTU’s representative sequence by Ribosomal Database Project (RDP) 16S classifier (72). Singletons were removed for downstream analyses. To minimize the impact of read count variation from different samples, we rarefied all samples based on the smallest sequence numbers (20,000 sequences). The above-mentioned steps were performed through the Galaxy pipeline at the Institute for Environmental Genomics, University of Oklahoma (http://zhoulab5.rccc.ou.edu:8080/root/login?redirect=%2F). Al-tolerant bacterial communities were identified according to the microorganisms carrying Al-resistant functional genes detected by GeoChip 5.0. The source organisms of Al-resistant functional genes detected by GeoChip 5.0 were considered potential Al-tolerant microorganisms. We then identified these potential Al-tolerant microorganisms from the 16S detected taxa as Al-tolerant taxa.

### GeoChip 5.0 hybridization.

GeoChip 5.0 is a functional gene array that contains 346 and 1,668 specific probes covering genes for Al resistance and antioxidant enzyme, respectively (36). We used GeoChip 5.0 to examine microbial functional potentials, including Al resistance and antioxidant enzyme. An aliquot of 1 μg of DNA from each sample was labeled directly, purified, and resuspended in 50 μl of hybridization solution that contained 10% formamide, 5× SSC (1× SSC is 0.15 M NaCl plus 0.015 M sodium citrate), 0.1% SDS, 0.1 mg/ml of salmon sperm DNA, and 2 μl of common oligonucleotide reference standard (0.1 pmol/μl) (73, 74). The fluorescently labeled DNA was hybridized with GeoChip 5.0 on a MAUI hybridization system (BioMicro Systems, UT, USA) at 65°C for 12 h. The microarrays were scanned using a ScanArray 5000 microarray analysis system (PerkinElmer, Wellesley, MA, USA) at 95% laser power and 68% photomultiplier tube gain. Scanned images were saved in 16-bit TIFF format, quantified using ImaGene version 6.0 (BioDiscovery, Inc., Los Angeles, CA, USA), and processed in the Microarray Data Manager system at the Institute for Environmental Genomics website (http://ieg.ou.edu/microarray). Spots with signal-to-noise ratios lower than 2.0 were removed before statistical analysis, as previously described (75).

### Statistical analyses.

The alpha diversity (richness and Shannon index) of each sample was calculated, and the beta diversity was estimated based on the Bray-Curtis distance between samples. The geographical distances among the sampling sites were calculated based on the sampling coordinates. Redundancy analysis (RDA) was performed using the rda function in the vegan R package to examine the relationships between the Al-tolerant bacterial community and major climatic and edaphic variables. Climatic and edaphic variables were fitted with the ordination plots using vegan R package with 9,999 permutations, and the biplot was displayed with scaling of 2. A forward selection procedure was applied to select significant variables (*P < *0.05, with 999 permutations) with the ordiR2step function of the vegan R package (76, 77). Partial Mantel analysis was carried out to determine the contributions of various environmental factors to explain the variation of Al-tolerant bacterial OTUs. The distances of environmental variables and Al-tolerant bacterial community among samples were calculated based on Euclidean dissimilarity and Bray-Curtis dissimilarity, respectively. Partial mantel tests were performed with Pearson correlations and 1,000 permutations using the mantel function in ecodist R package (78). Spearman correlations between the abundance of Al-resistant genes and antioxidant genes were analyzed using the cor.test function in R.

A directed network is established to elucidate asymmetric underlying relationships among community members, which can be used to infer the directionality of information flows in ecological networks (33–35). A classification scheme for assessing the relative importance of different ecological processes based on time series was proposed by Faust et al. (79). The scheme is based on a test of the temporal structure in a given time series via analyses of noise type profile and neutrality. We used this approach to determine the ecological drivers of microbial dynamics to guide the selection of appropriate community models for prediction and follow-up analysis. First, 144 soil samples with pH <5.1 were selected to form a set of continuous data with equal steps (ΔpH = 0.01). It was then determined whether dependence between successive pH steps occurred in the pH series. The dependency was measured by noise types of species, which can be distinguished as black, brown, pink, and white noise. A shift in the noise color from black to pink indicates a reduced dependency on community within the pH series, while white noise indicates the absence of any dependency. In brief, the dependency on community between pH steps is strongest for black noise and weakest for pink noise, while it is absent for white noise. Four types of noise results revealed that the presence of non-white noise types was a robust indicator of this successive structure. Second, we tested the structured models at a *P *value of >0.5 for neutrality and determined the goodness of fit against a deterministic model. The structure has a good fit with the Ricker model. Finally, interactions were inferred from the pH <5.1 data sets, and the inferred association matrices were represented as directed networks. Links between bacteria were counted as the number of entries in the interaction matrix. The significance of link number was assessed by repeatedly (100 times) randomizing the interaction matrix while preserving the total number of entries and computing parameter-free *P* values. According to the magnitude of the in-degree (the number of associations pointing to the node, indicating the degree of the species affected by others) or out-degree (the number of associations pointing out of the node, indicating the degree of the species affecting others) between species in the directed network, we divided the species into influential taxa that only affect others (out-degree only), sensitive taxa that are only affected by others (in-degree only), and bidirectional taxa that affect and are affected by others (both out-degree and in-degree). These analyses were performed with the seqtime R package (https://github.com/hallucigenia-sparsa/seqtime) (79). The networks were visualized on the interactive Gephi platform (80). Nodes represent the individual species, and directed edges represent the significant associations (*P < *0.05) between the nodes in the network, indicating their potential interactive relationships. The layout was displayed using the Fruchterman-Reingold algorithm.

We generated a nonmetric multidimensional scaling (NMDS) ordination of a community of the three taxa based on the Bray-Curtis dissimilarity matrix. We employed the ordisurf function within the vegan R package to fit trait data to the Al-tolerant bacterial community ordination using generalized additive models (GAMs). These models fitted the environmental data as a smooth response surface over the three taxa of community ordinations accounting for both NMDS axes. To determine the relative importance of environmental factors in structuring the three taxa of bacterial communities, we conducted multiple regression analysis using the MRM approach. Because there was strong collinearity among particular environmental factors, we employed variable clustering before applying MRM to assess the redundancy of the environmental variables by the varclus procedure of the Hmisc R package (81). Any variables with high correlation (Spearman’s ρ^2^ > 0.7) were removed from the MRM analysis (for example, NO_3_-N and DTN); all other variables were retained in the models. We then implemented a matrix randomization procedure with standardized predictor variables using ecodist R package (78). To account for zero-similarity values, bacterial community dissimilarity (the Bray-Curtis distance) was ln transformed (82).

To help explain the variation in the composition of the three taxa, we estimated Levins’ niche breadth (*B*) index (83) for the microbial taxa members according to the following equation:

Bj=1∑i=1NPij2

where *B_j_* denotes the habitat niche breadth of OTU*_j_* in a community, *N* is the total number of communities in each metacommunity, and *P_ij_* is the proportion of OTU *j* in community *i*. A high *B* value for a given OTU indicates a wide habitat niche breadth. The community-level *B* value (Bcom) was calculated as the average *B* value for all members in a community. We expect a microbial taxon with a wider niche breadth to be more metabolically flexible at the community level. The analysis was conducted with the niche.width function of the spaa R package (84).

To test for correlations between antioxidant functional genes and the three microbial taxa, we performed simple Mantel tests with 9,999 permutations using the mantel function within the vegan R package. All of the analyses were conducted in R version 3.7.1 (https://www.r-project.org/).

### Data availability.

Raw sequence data for 16S rRNA gene amplicons were deposited in the National Center for Biotechnology Information (NCBI) BioProject under accession no. PRJNA562601. The GeoChip data are available in the figshare repository, https://doi.org/10.6084/m9.figshare.9746303. The sampling information and soil geochemical data are also available in the figshare repository, https://doi.org/10.6084/m9.figshare.11493081.v2.
