# Supplementary figures and images for: Soil pH Filters the Association Patterns of Aluminum-Tolerant Microorganisms in Rice Paddies
Source: mSystems. 2022 Feb 15;7(1):e01022-21. doi: 10.1128/msystems.01022-21 (PMC8845571; doi:10.1128/msystems.01022-21)

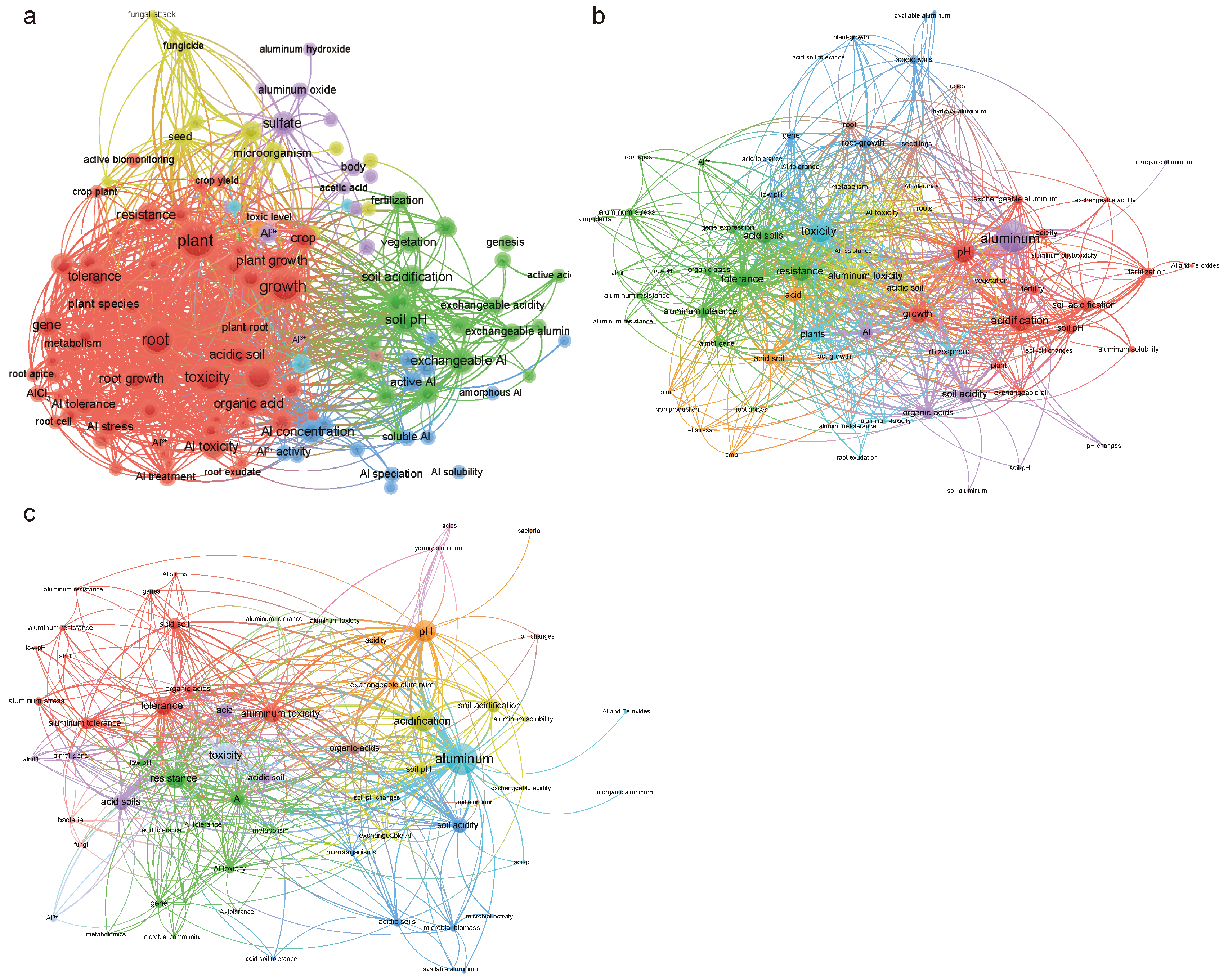

Supplement: FIG S1 [file msystems.01022-21-sf001.tif]

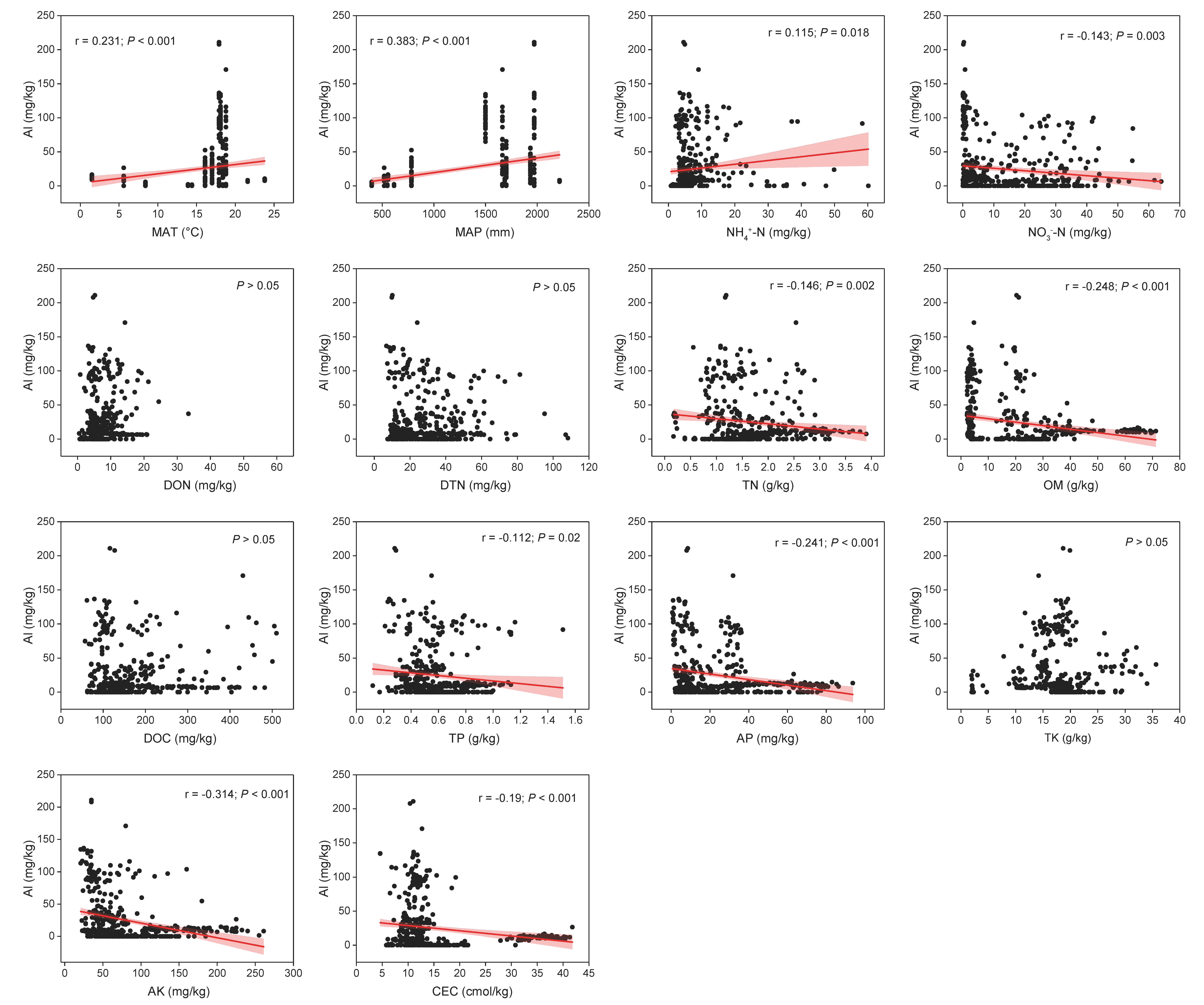

Supplement: FIG S2 [file msystems.01022-21-sf002.tif]

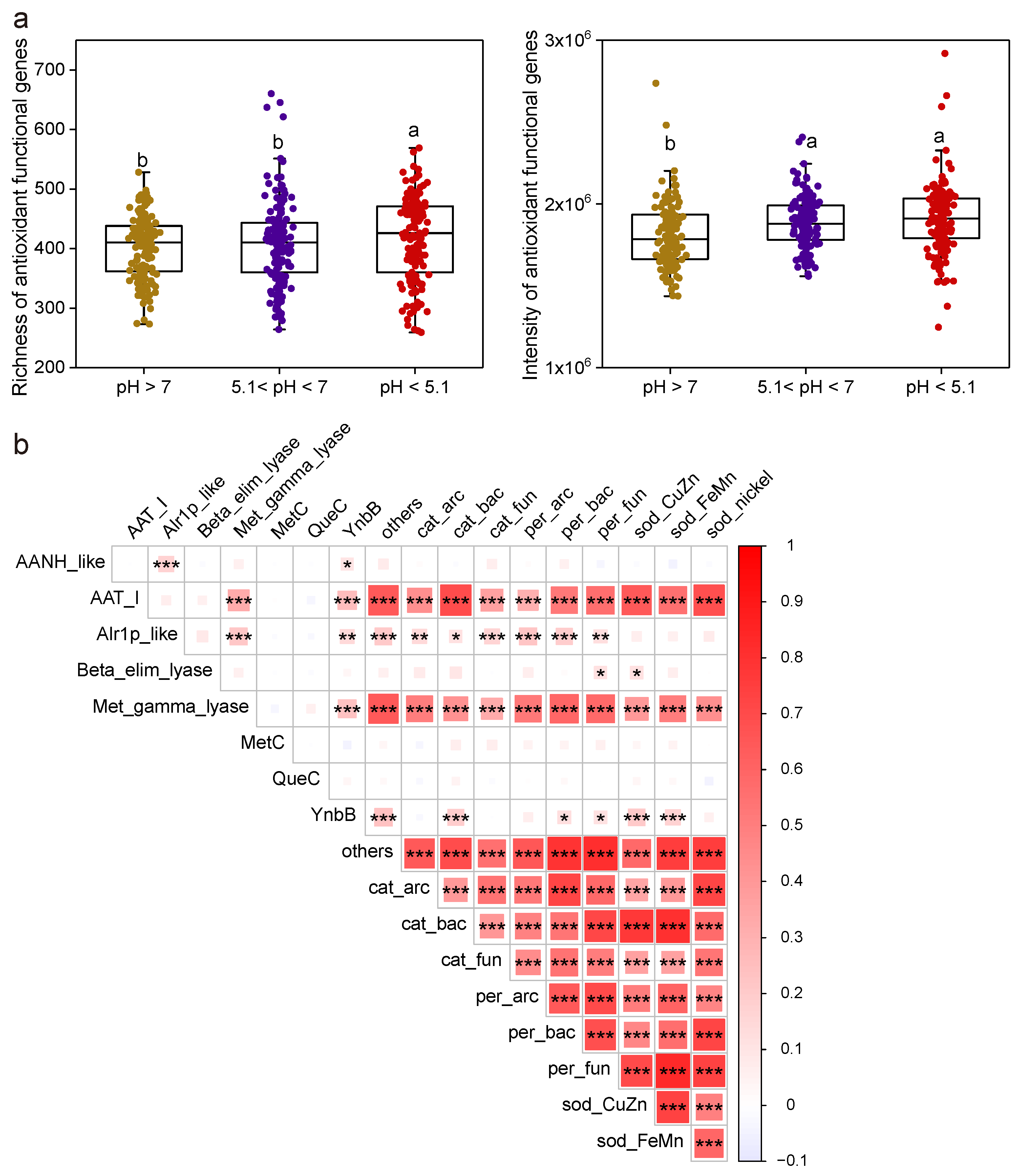

Supplement: FIG S3 [file msystems.01022-21-sf003.tif]

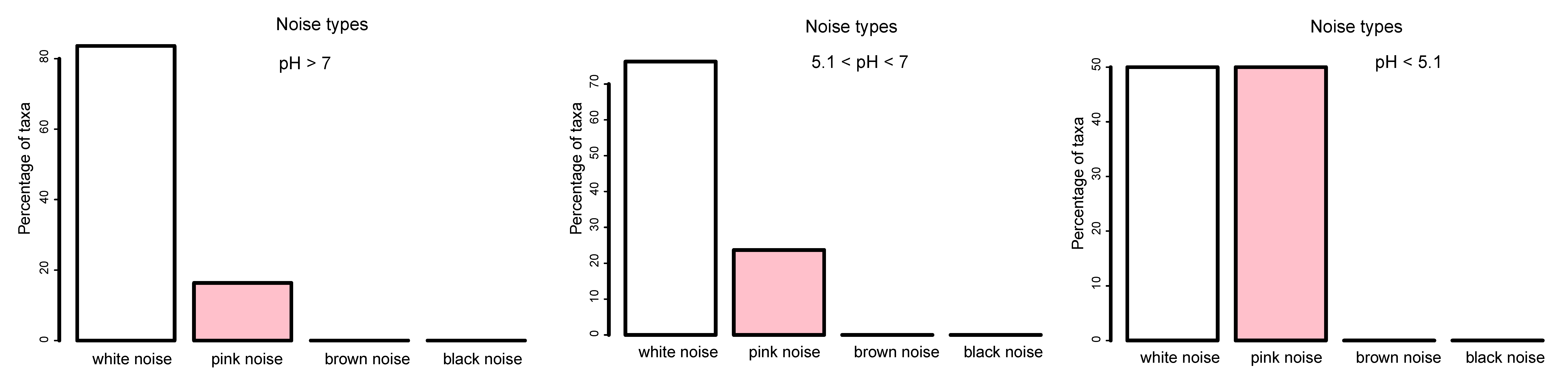

Supplement: FIG S4 [file msystems.01022-21-sf004.tif]

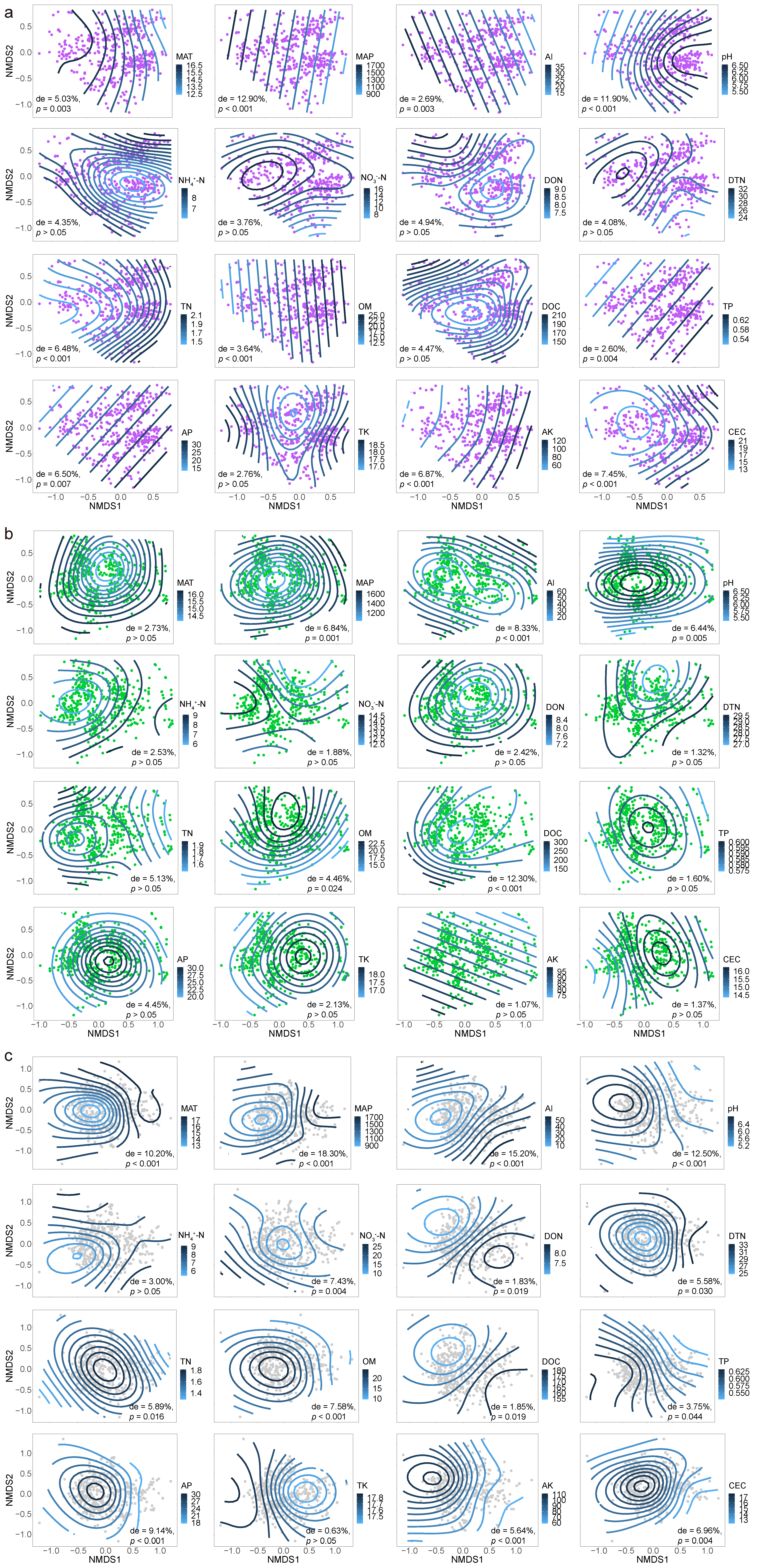

Supplement: FIG S5 [file msystems.01022-21-sf005.tif]
