# Supplementary material for: Soil pH Filters the Association Patterns of Aluminum-Tolerant Microorganisms in Rice Paddies
Source: mSystems. 2022 Feb 15;7(1):e01022-21. doi: 10.1128/msystems.01022-21 (PMC8845571; doi:10.1128/msystems.01022-21)
Supplement: TABLE S1 [file msystems.01022-21-st001.docx]

**Table S1** Al-resistant functional genes that encode Al-resistance proteins detected by GeoChip 5.0 assay.

| **Region name** | **Note** | **Number of probes identified by GeoChip 5.0** |
| --- | --- | --- |
| AANH_like | Adenine nucleotide alpha hydrolases superfamily including N type ATP PPases, ATP sulphurylases Universal Stress Response protein and electron transfer flavoprotein (ETF). | 4 |
| AAT_Ⅰ | Aspartate aminotransferase (AAT) superfamily (fold type I) of pyridoxal phosphate (PLP)-dependent enzymes. | 80 |
| Alr1p_like | Saccharomyces cerevisiae Alr1p-like subfamily. | 4 |
| Beta_elim_lyase | Beta-eliminating lyase; pfam01212. | 3 |
| Met_gamma_lyase | Methionine gamma-lyase; pfam06838. | 19 |
| MetC | cystathionine beta-lyase / L-cysteine desulfhydrase / alanine racemase. | 1 |
| QueC | Queuosine biosynthesis protein QueC; pfam06508. | 1 |
| YnbB | Cystathionine beta-lyase family protein involved in aluminum resistance [Inorganic ion transport and metabolism, General function prediction only]. | 9 |
| others | / | 112 |
