# Supplementary material for: Soil pH Filters the Association Patterns of Aluminum-Tolerant Microorganisms in Rice Paddies
Source: mSystems. 2022 Feb 15;7(1):e01022-21. doi: 10.1128/msystems.01022-21 (PMC8845571; doi:10.1128/msystems.01022-21)
Supplement: TABLE S2 [file msystems.01022-21-st002.docx]

**Table S2** Taxonomic information of source organisms of Al-resistant functional genes detected by GeoChip 5.0.

| **Gene** | **Source organisms** | | | | |
| --- | --- | --- | --- | --- | --- |
|  | **phylum** | **class** | **order** | **family** | **genus** |
| Al-resistant gene | Caldiserica | Caldisericia | Caldisericales | Caldisericaceae | *Caldisericum* |
| Al-resistant gene | Cyanobacteria | Gloeobacteria | Gloeobacterales |  | *Gloeobacter* |
| Al-resistant gene | Cyanobacteria |  | Chroococcales |  | *Acaryochloris* |
| Al-resistant gene | Cyanobacteria |  | Chroococcales |  | *Cyanothece* |
| Al-resistant gene | Cyanobacteria |  | Chroococcales |  | *Synechococcus* |
| Al-resistant gene | Cyanobacteria |  | Nostocales | Nostocaceae | *Nodularia* |
| Al-resistant gene | Cyanobacteria |  | Nostocales | Nostocaceae | *Nostoc* |
| Al-resistant gene | Cyanobacteria |  | Nostocales | Nostocaceae | *Raphidiopsis* |
| Al-resistant gene | Cyanobacteria |  | Oscillatoriales |  | *Arthrospira* |
| Al-resistant gene | Cyanobacteria |  | Oscillatoriales |  | *Microcoleus* |
| Al-resistant gene | Cyanobacteria |  | Oscillatoriales |  | *Moorea* |
| Al-resistant gene | Cyanobacteria |  | Oscillatoriales |  | *Oscillatoria* |
| Al-resistant gene | Cyanobacteria |  | Prochlorales | Prochlorococcaceae | *Prochlorococcus* |
| Al-resistant gene | Cyanobacteria |  | Stigonematales |  | *Fischerella* |
| Al-resistant gene | Firmicutes | Bacilli | Bacillales | Alicyclobacillaceae | *Kyrpidia* |
| Al-resistant gene | Firmicutes | Bacilli | Bacillales | Bacillaceae | *Bacillus* |
| Al-resistant gene | Firmicutes | Bacilli | Bacillales | Bacillaceae | *Caldalkalibacillus* |
| Al-resistant gene | Firmicutes | Bacilli | Bacillales | Bacillaceae | *Geobacillus* |
| Al-resistant gene | Firmicutes | Bacilli | Bacillales | Bacillaceae | *Halobacillus* |
| Al-resistant gene | Firmicutes | Bacilli | Bacillales | Bacillaceae | *Lentibacillus* |
| Al-resistant gene | Firmicutes | Bacilli | Bacillales | Bacillaceae | *Lysinibacillus* |
| Al-resistant gene | Firmicutes | Bacilli | Bacillales | Listeriaceae | *Listeria* |
| Al-resistant gene | Firmicutes | Bacilli | Bacillales | Paenibacillaceae | *Paenibacillus* |
| Al-resistant gene | Firmicutes | Bacilli | Bacillales | Paenibacillaceae | *Thermobacillus* |
| Al-resistant gene | Firmicutes | Bacilli | Bacillales | Planococcaceae | *Planococcus* |
| Al-resistant gene | Firmicutes | Bacilli | Bacillales | Planococcaceae | *Solibacillus* |
| Al-resistant gene | Firmicutes | Bacilli | Bacillales | Planococcaceae | *Sporosarcina* |
| Al-resistant gene | Firmicutes | Bacilli | Bacillales | Sporolactobacillaceae | *Sporolactobacillus* |
| Al-resistant gene | Firmicutes | Bacilli | Bacillales | Staphylococcaceae | *Staphylococcus* |
| Al-resistant gene | Firmicutes | Bacilli | Bacillales | Thermoactinomycetaceae | *Desmospora* |
| Al-resistant gene | Firmicutes | Bacilli | Bacillales |  | *Exiguobacterium* |
| Al-resistant gene | Firmicutes | Bacilli | Lactobacillales | Aerococcaceae | *Abiotrophia* |
| Al-resistant gene | Firmicutes | Bacilli | Lactobacillales | Lactobacillaceae | *Lactobacillus* |
| Al-resistant gene | Firmicutes | Bacilli | Lactobacillales | Lactobacillaceae | *Pediococcus* |
| Al-resistant gene | Firmicutes | Clostridia | Clostridiales | Clostridiaceae | *Alkaliphilus* |
| Al-resistant gene | Firmicutes | Clostridia | Clostridiales | Clostridiaceae | *Clostridium* |
| Al-resistant gene | Firmicutes | Clostridia | Clostridiales | Clostridiales Family XI. Incertae Sedis | *Anaerococcus* |
| Al-resistant gene | Firmicutes | Clostridia | Clostridiales | Clostridiales Family XI. Incertae Sedis | *Finegoldia* |
| Al-resistant gene | Firmicutes | Clostridia | Clostridiales | Clostridiales Family XI. Incertae Sedis | *Parvimonas* |
| Al-resistant gene | Firmicutes | Clostridia | Clostridiales | Clostridiales Family XI. Incertae Sedis | *Peptoniphilus* |
| Al-resistant gene | Firmicutes | Clostridia | Clostridiales | Clostridiales Family XIII. Incertae Sedis |  |
| Al-resistant gene | Firmicutes | Clostridia | Clostridiales | Clostridiales Family XVIII. Incertae Sedis | *Symbiobacterium* |
| Al-resistant gene | Firmicutes | Clostridia | Clostridiales | Eubacteriaceae | *Anaerofustis* |
| Al-resistant gene | Firmicutes | Clostridia | Clostridiales | Eubacteriaceae | *Eubacterium* |
| Al-resistant gene | Firmicutes | Clostridia | Clostridiales | Eubacteriaceae | *Pseudoramibacter* |
| Al-resistant gene | Firmicutes | Clostridia | Clostridiales | Heliobacteriaceae | *Heliobacterium* |
| Al-resistant gene | Firmicutes | Clostridia | Clostridiales | Lachnospiraceae | *Blautia* |
| Al-resistant gene | Firmicutes | Clostridia | Clostridiales | Lachnospiraceae | *Butyrivibrio* |
| Al-resistant gene | Firmicutes | Clostridia | Clostridiales | Lachnospiraceae | *Cellulosilyticum* |
| Al-resistant gene | Firmicutes | Clostridia | Clostridiales | Lachnospiraceae | *Coprococcus* |
| Al-resistant gene | Firmicutes | Clostridia | Clostridiales | Lachnospiraceae | *Johnsonella* |
| Al-resistant gene | Firmicutes | Clostridia | Clostridiales | Lachnospiraceae | *Lachnoanaerobaculum* |
| Al-resistant gene | Firmicutes | Clostridia | Clostridiales | Lachnospiraceae | *Marvinbryantia* |
| Al-resistant gene | Firmicutes | Clostridia | Clostridiales | Lachnospiraceae | *Oribacterium* |
| Al-resistant gene | Firmicutes | Clostridia | Clostridiales | Lachnospiraceae | *Roseburia* |
| Al-resistant gene | Firmicutes | Clostridia | Clostridiales | Lachnospiraceae | *Shuttleworthia* |
| Al-resistant gene | Firmicutes | Clostridia | Clostridiales | Lachnospiraceae | *Stomatobaculum* |
| Al-resistant gene | Firmicutes | Clostridia | Clostridiales | Oscillospiraceae | *Oscillibacter* |
| Al-resistant gene | Firmicutes | Clostridia | Clostridiales | Peptococcaceae | *Candidatus Desulforudis* |
| Al-resistant gene | Firmicutes | Clostridia | Clostridiales | Peptococcaceae | *Desulfitobacterium* |
| Al-resistant gene | Firmicutes | Clostridia | Clostridiales | Peptococcaceae | *Desulfosporosinus* |
| Al-resistant gene | Firmicutes | Clostridia | Clostridiales | Peptococcaceae | *Desulfotomaculum* |
| Al-resistant gene | Firmicutes | Clostridia | Clostridiales | Peptococcaceae | *Syntrophobotulus* |
| Al-resistant gene | Firmicutes | Clostridia | Clostridiales | Peptococcaceae | *Thermincola* |
| Al-resistant gene | Firmicutes | Clostridia | Clostridiales | Peptostreptococcaceae | *Peptostreptococcus* |
| Al-resistant gene | Firmicutes | Clostridia | Clostridiales | Ruminococcaceae | *Anaerotruncus* |
| Al-resistant gene | Firmicutes | Clostridia | Clostridiales | Ruminococcaceae | *Ethanoligenens* |
| Al-resistant gene | Firmicutes | Clostridia | Clostridiales | Ruminococcaceae | *Ruminococcus* |
| Al-resistant gene | Firmicutes | Clostridia | Clostridiales | Ruminococcaceae | *Subdoligranulum* |
| Al-resistant gene | Firmicutes | Clostridia | Clostridiales |  | *Epulopiscium* |
| Al-resistant gene | Firmicutes | Clostridia | Clostridiales |  | *Pseudoflavonifractor* |
| Al-resistant gene | Firmicutes | Clostridia | Clostridiales | Syntrophomonadaceae | *Dethiobacter* |
| Al-resistant gene | Firmicutes | Clostridia | Natranaerobiales | Natranaerobiaceae | *Natranaerobius* |
| Al-resistant gene | Firmicutes | Clostridia | Thermoanaerobacterales | Thermoanaerobacteraceae | *Ammonifex* |
| Al-resistant gene | Firmicutes | Clostridia | Thermoanaerobacterales | Thermoanaerobacteraceae | *Caldanaerobacter* |
| Al-resistant gene | Firmicutes | Clostridia | Thermoanaerobacterales | Thermoanaerobacteraceae | *Carboxydothermus* |
| Al-resistant gene | Firmicutes | Clostridia | Thermoanaerobacterales | Thermoanaerobacteraceae | *Moorella* |
| Al-resistant gene | Firmicutes | Clostridia | Thermoanaerobacterales | Thermoanaerobacterales Family III. Incertae Sedis | *Caldicellulosiruptor* |
| Al-resistant gene | Firmicutes | Clostridia | Thermoanaerobacterales | Thermoanaerobacterales Family III. Incertae Sedis | *Thermoanaerobacterium* |
| Al-resistant gene | Firmicutes | Clostridia | Thermoanaerobacterales | Thermoanaerobacterales Family III. Incertae Sedis | *Thermosediminibacter* |
| Al-resistant gene | Firmicutes | Erysipelotrichia | Erysipelotrichales | Erysipelotrichaceae | *Bulleidia* |
| Al-resistant gene | Firmicutes | Negativicutes | Selenomonadales | Acidaminococcaceae | *Acidaminococcus* |
| Al-resistant gene | Firmicutes | Negativicutes | Selenomonadales | Acidaminococcaceae | *Phascolarctobacterium* |
| Al-resistant gene | Firmicutes | Negativicutes | Selenomonadales | Veillonellaceae | *Acetonema* |
| Al-resistant gene | Firmicutes | Negativicutes | Selenomonadales | Veillonellaceae | *Centipeda* |
| Al-resistant gene | Firmicutes | Negativicutes | Selenomonadales | Veillonellaceae | *Dialister* |
| Al-resistant gene | Firmicutes | Negativicutes | Selenomonadales | Veillonellaceae | *Megamonas* |
| Al-resistant gene | Firmicutes | Negativicutes | Selenomonadales | Veillonellaceae | *Megasphaera* |
| Al-resistant gene | Firmicutes | Negativicutes | Selenomonadales | Veillonellaceae | *Selenomonas* |
| Al-resistant gene | Firmicutes | Negativicutes | Selenomonadales | Veillonellaceae | *Thermosinus* |
| Al-resistant gene | Firmicutes | Negativicutes | Selenomonadales | Veillonellaceae | *Veillonella* |
| Al-resistant gene | Proteobacteria | Gammaproteobacteria | Pasteurellales | Pasteurellaceae | *Actinobacillus* |
