# Supplementary material for: Soil pH Filters the Association Patterns of Aluminum-Tolerant Microorganisms in Rice Paddies
Source: mSystems. 2022 Feb 15;7(1):e01022-21. doi: 10.1128/msystems.01022-21 (PMC8845571; doi:10.1128/msystems.01022-21)
Supplement: TABLE S3 [file msystems.01022-21-st003.docx]

**Table** **S3** Partial Mantel analysis of the relationship between the environmental variables and Al-tolerant bacterial community.

| **variable** | **control** | **r** | ***p*** |
| --- | --- | --- | --- |
| **MAT** | **Others** | **0.17** | **0.004** |
| **MAP** | **Others** | **0.34** | **0.001** |
| **pH** | **Others** | **0.35** | **0.001** |
| **exchangeable Al^3+^** | **Others** | **0.17** | **0.009** |
| NH_4_^+^-N | Others | -0.05 | >0.05 |
| **NO_3_^-^-N** | **Others** | **0.22** | **0.001** |
| DON | Others | -0.04 | >0.05 |
| DTN | Others | -0.13 | >0.05 |
| TN | Others | -0.12 | >0.05 |
| OM | Others | 0.06 | >0.05 |
| **DOC** | **Others** | **0.09** | **0.041** |
| TP | Others | -0.12 | >0.05 |
| AP | Others | -0.02 | >0.05 |
| TK | Others | -0.05 | >0.05 |
| **AK** | **Others** | **0.14** | **0.011** |
| CEC | Others | -0.15 | >0.05 |

The values in bold font indicate significant correlations at *p* < 0.05.MAT: mean annual temperature; MAP: mean annual precipitation; DON: dissolved organic nitrogen; DTN: dissolve total nitrogen; TN: total nitrogen; OM: organic matter; DOC: dissolved organic carbon; TP: total phosphorus; AP: available phosphorus; TK: total potassium; AK: available potassium; CEC: cation exchange capacity.
