# Supplementary material for: Soil pH Filters the Association Patterns of Aluminum-Tolerant Microorganisms in Rice Paddies
Source: mSystems. 2022 Feb 15;7(1):e01022-21. doi: 10.1128/msystems.01022-21 (PMC8845571; doi:10.1128/msystems.01022-21)
Supplement: TABLE S4 [file msystems.01022-21-st004.docx]

**Table S4** Antioxidant functional genes on GeoChip 5.0 array.

| **Subcategory** | **Gene** | **Encoded enzyme** | **Number of probes on GeoChip 5.0 assay** |
| --- | --- | --- | --- |
| Antioxidant enzyme | cat_arc | Catalase | 10 |
| Antioxidant enzyme | cat_bac | Catalase | 461 |
| Antioxidant enzyme | cat_fun | Peroxisomal catalase | 105 |
| Antioxidant enzyme | per_arc | Alkyl hydroperoxide reductase / Thiol specific antioxidant / Mal allergen | 22 |
| Antioxidant enzyme | per_bac | Peroxidase | 33 |
| Antioxidant enzyme | per_fun | Versatile peroxidase VPL1 | 177 |
| Antioxidant enzyme | sod_CuZn | Superoxide dismutase [Cu-Zn] precursor | 137 |
| Antioxidant enzyme | sod_FeMn | Mn-dependent superoxide dismutase | 699 |
| Antioxidant enzyme | sod_nickel | Putative nickel-containing superoxide dismutase precursor (NISOD) | 24 |
