# Supplementary material for: Soil pH Filters the Association Patterns of Aluminum-Tolerant Microorganisms in Rice Paddies
Source: mSystems. 2022 Feb 15;7(1):e01022-21. doi: 10.1128/msystems.01022-21 (PMC8845571; doi:10.1128/msystems.01022-21)
Supplement: TABLE S5 [file msystems.01022-21-st005.docx]

**Table S5** Classification of three taxa based on the out- and in-degrees of the directed network in acidic soils (pH < 5.1).

|  | OTUs | Out | In | Out/In | Domain | Phylum | Class | Order | Family | Genus |
| --- | --- | --- | --- | --- | --- | --- | --- | --- | --- | --- |
| Influential taxa | OTU_3153 | 4 | / | / | Bacteria | Firmicutes | Clostridia | Clostridiales | Ruminococcaceae | *Clostridium* III |
| Influential taxa | OTU_45175 | 3 | / | / | Bacteria | Firmicutes | Clostridia | Clostridiales | Ruminococcaceae | *Clostridium* III |
| Influential taxa | OTU_2033 | 2 | / | / | Bacteria | Firmicutes | Clostridia | Clostridiales | Peptococcaceae | *Desulfosporosinus* |
| Influential taxa | OTU_4037 | 2 | / | / | Bacteria | Firmicutes | Clostridia | Clostridiales | Ruminococcaceae | *Clostridium* III |
| Influential taxa | OTU_12 | 1 | / | / | Bacteria | Firmicutes | Bacilli | Bacillales | Bacillaceae | *Bacillus* |
| Influential taxa | OTU_1299 | 1 | / | / | Bacteria | Firmicutes | Clostridia | Clostridiales | Peptostreptococcaceae | *Clostridium* XI |
| Influential taxa | OTU_2572 | 1 | / | / | Bacteria | Firmicutes | Clostridia | Clostridiales | Peptostreptococcaceae | *Clostridium* XI |
| Influential taxa | OTU_2779 | 1 | / | / | Bacteria | Firmicutes | Bacilli | Bacillales | Paenibacillaceae | *Paenibacillus* |
| Influential taxa | OTU_35454 | 1 | / | / | Bacteria | Firmicutes | Bacilli | Bacillales | Bacillaceae | *Bacillus* |
| Influential taxa | OTU_44225 | 1 | / | / | Bacteria | Firmicutes | Clostridia | Clostridiales | Peptostreptococcaceae | *Clostridium* XI |
| Influential taxa | OTU_945 | 1 | / | / | Bacteria | Firmicutes | Bacilli | Bacillales | Paenibacillaceae | *Paenibacillus* |
| Sensitive taxa | OTU_1601 | / | 3 | / | Bacteria | Firmicutes | Bacilli | Bacillales | Paenibacillaceae | *Paenibacillus* |
| Sensitive taxa | OTU_11371 | / | 2 | / | Bacteria | Firmicutes | Clostridia | Clostridiales | Ruminococcaceae | *Clostridium* III |
| Sensitive taxa | OTU_2900 | / | 2 | / | Bacteria | Firmicutes | Bacilli | Bacillales | Bacillaceae | *Bacillus* |
| Sensitive taxa | OTU_106 | / | 1 | / | Bacteria | Firmicutes | Bacilli | Bacillales | Bacillaceae | *Bacillus* |
| Sensitive taxa | OTU_126 | / | 1 | / | Bacteria | Firmicutes | Clostridia | Clostridiales | Peptostreptococcaceae | *Clostridium* XI |
| Sensitive taxa | OTU_14296 | / | 1 | / | Bacteria | Firmicutes | Clostridia | Clostridiales | Ruminococcaceae | *Clostridium* III |
| Sensitive taxa | OTU_16461 | / | 1 | / | Bacteria | Firmicutes | Bacilli | Bacillales | Bacillaceae | *Bacillus* |
| Sensitive taxa | OTU_26774 | / | 1 | / | Bacteria | Firmicutes | Clostridia | Clostridiales | Lachnospiraceae | *Clostridium* XlVa |
| Sensitive taxa | OTU_34533 | / | 1 | / | Bacteria | Firmicutes | Bacilli | Bacillales | Bacillaceae | *Bacillus* |
| Bidirectional taxa | OTU_13122 | 2 | 1 | 2 | Bacteria | Firmicutes | Clostridia | Clostridiales | Ruminococcaceae | *Clostridium* III |
| Bidirectional taxa | OTU_1957 | 2 | 1 | 2 | Bacteria | Firmicutes | Bacilli | Bacillales | Paenibacillaceae | *Paenibacillus* |
| Bidirectional taxa | OTU_30702 | 3 | 2 | 1.5 | Bacteria | Firmicutes | Clostridia | Clostridiales | Ruminococcaceae | *Clostridium* III |
| Bidirectional taxa | OTU_170 | 1 | 1 | 1 | Bacteria | Firmicutes | Clostridia | Clostridiales | Peptostreptococcaceae | *Clostridium* XI |
| Bidirectional taxa | OTU_1743 | 1 | 1 | 1 | Bacteria | Firmicutes | Clostridia | Clostridiales | Ruminococcaceae | *Clostridium* III |
| Bidirectional taxa | OTU_2168 | 1 | 1 | 1 | Bacteria | Firmicutes | Clostridia | Clostridiales | Peptococcaceae | *Desulfosporosinus* |
| Bidirectional taxa | OTU_2294 | 1 | 1 | 1 | Bacteria | Firmicutes | Bacilli | Bacillales | Paenibacillaceae | *Paenibacillus* |
| Bidirectional taxa | OTU_25197 | 1 | 1 | 1 | Bacteria | Firmicutes | Bacilli | Bacillales | Paenibacillaceae | *Paenibacillus* |
| Bidirectional taxa | OTU_3549 | 1 | 1 | 1 | Bacteria | Firmicutes | Bacilli | Bacillales | Bacillaceae | *Bacillus* |
| Bidirectional taxa | OTU_46880 | 1 | 1 | 1 | Bacteria | Firmicutes | Clostridia | Clostridiales | Ruminococcaceae | *Clostridium* III |
| Bidirectional taxa | OTU_7112 | 1 | 1 | 1 | Bacteria | Firmicutes | Clostridia | Clostridiales | Peptococcaceae | *Desulfitobacterium* |
| Bidirectional taxa | OTU_4525 | 1 | 2 | 0.5 | Bacteria | Firmicutes | Bacilli | Bacillales | Paenibacillaceae | *Paenibacillus* |
| Bidirectional taxa | OTU_7118 | 2 | 4 | 0.5 | Bacteria | Firmicutes | Clostridia | Clostridiales | Peptococcaceae | *Desulfitobacterium* |
| Bidirectional taxa | OTU_27686 | 1 | 3 | 0.33 | Bacteria | Firmicutes | Clostridia | Clostridiales | Ruminococcaceae | *Clostridium* III |
| Bidirectional taxa | OTU_2510 | 1 | 4 | 0.25 | Bacteria | Firmicutes | Clostridia | Clostridiales | Peptostreptococcaceae | *Clostridium* XI |
